# FastRet: Fast and Simple Retention Time Prediction in Liquid Chromatography

**DOI:** 10.1021/acs.jcim.6c01344

**Published:** 2026-08-12

**Authors:** Fadi Fadil, Tobias Schmidt, Christian Amesoeder, Simon Heckscher, Marian Schoen, Wolfram Gronwald, Peter J. Oefner, Rainer Spang, Katja Dettmer

**Affiliations:** † Institute of Functional Genomics, 9147 University of Regensburg, 93053 Regensburg, Germany; ‡ Institute of Medical Microbiology, Immunology and Hygiene, University Hospital Cologne, 50935 Köln, Germany; § Department of Statistical Bioinformatics, University of Regensburg, 93053 Regensburg, Germany

## Abstract

Feature annotation in liquid chromatography–mass spectrometry (LC–MS)-based untargeted metabolomics remains challenging. Retention time (RT) prediction can support candidate prioritization and improve annotation confidence. Here, we present FastRet, an R package predicting RTs using Least Absolute Shrinkage and Selection Operator (LASSO) and Boosted Regression Trees (BRT) on molecular descriptors. FastRet provides a flexible framework combining from-scratch model training, selective measuring to prioritize metabolites for remeasurement, and model adjustment to adapt existing models to changed chromatographic conditions. Model training and prediction are completed within seconds on a single CPU core, and FastRet is accessible both from the R console and through a web interface. We validated FastRet on three in-house data sets covering reversed-phase chromatography (RP; *N* = 458), RP–anion-exchange mixed-mode chromatography (RP-AXMM; *N* = 436), and hydrophilic interaction chromatography (HILIC; *N* = 388), plus one external HILIC data set from the Retip package (*N* = 970). Using a 2:1 training/test split, BRT models trained from scratch achieved a test-set coefficient of determination (*R* ^2^) of 0.86, 0.66, and 0.81 for the three in-house data sets. FastRet can also adjust a model to new chromatographic conditions from a few remeasured metabolites: using 25 RP metabolites measured under six modified conditions, adjustment reached *R* ^2^ of 0.74 to 0.84 on unseen metabolites, a mean 0.22 gain over from-scratch models. Compared with published methods on identical splits, FastRet showed competitive performance for de novo prediction and superior performance in low-data transfer scenarios, while generalizing to 14 external data sets (median held-out *R* ^2^ 0.59). FastRet is available on CRAN with the web interface hosted at https://fastret.spang-lab.de.

## Graphical abstract

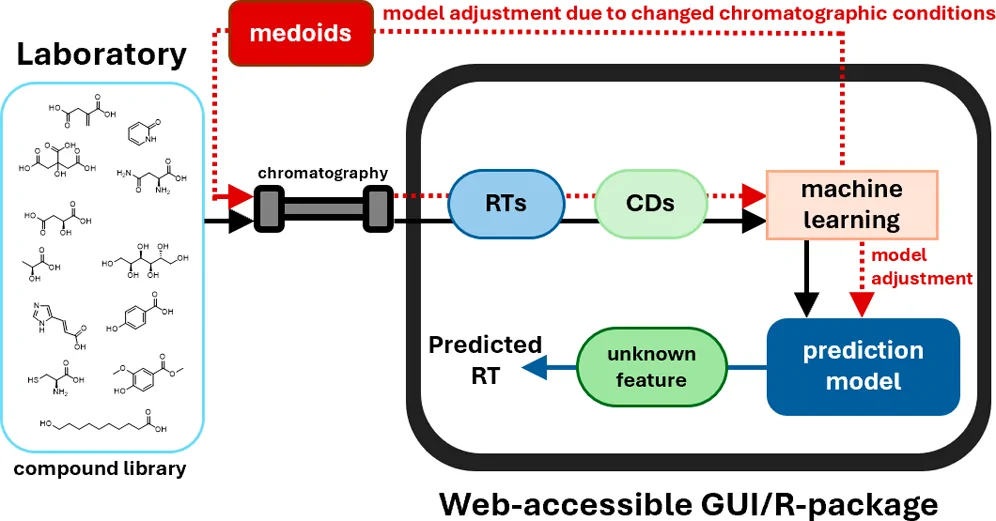

## Introduction

The metabolome holds a vast array of information that is thought to be most predictive of phenotype.^1^ LC–MS-based untargeted metabolomics requires the identification of unknown features to decipher metabolic changes. This task still poses a time-consuming challenge. In 2015, da Silva et al. concluded that only 1.8% of spectra in an untargeted metabolomics experiment can be annotated.^2^

As early as 2007, the metabolomics standards initiative established guidelines proposing four levels of identification confidence.^3^ Accordingly, level one identification requires two orthogonal data (e.g., RT and mass spectrum) relative to an authentic standard. A meta-analysis by Kodra et al. revealed that only 20% of the 1770 LC–MS metabolomics papers published in 2020 achieved level one identification.^4^ The main limiting factor of level one identification is the use of authentic standards. A tentative compound annotation can be achieved based solely on mass spectral information. Taking advantage of the RT, the second dimension of an LC–MS experiment, helps to further filter candidate annotations. However, unlike accurate masses and mass spectra, RTs cannot be simply retrieved from databases as they depend on numerous experimental factors, including stationary-phase properties (e.g., particle size and grafted functional groups), mobile-phase conditions (e.g., composition, gradient, and flow rate), column dimensions, temperature, and the LC system’s void volume. In particular, different separation mechanisms, such as RP-chromatography or HILIC, must be considered. Therefore, determining RTs for all candidate compounds would require measuring each compound on every LC system, which is neither practical nor cost-effective, especially as suitable standards may not always be commercially available. RT prediction can help to address these challenges by excluding potential candidates whose predicted RTs deviate considerably from the measured RT. The RT prediction problem addressed here is the estimation of RT for a fixed chromatographic setup from the molecular structure, represented by chemical descriptors. Although it may not yield definitive identifications, it can accelerate the annotation process and reduce experimental costs.

RT prediction for LC has been reviewed by Haddad et al.^5^ and Witting and Böcker,^6^ with Witting and Böcker focusing in particular on RT prediction in metabolomics. Haddad et al.^5^ categorize RT prediction strategies into physicochemical models and statistical models, respectively. While the former rely on mechanistic understanding of chromatographic processes, statistical models derive empirical relationships from data without requiring such insight. We focus on statistical models. Statistical models are built using either Experimental Design or Quantitative Structure–Retention Relationship (QSRR) approaches.^5^ Experimental design approaches use systematically collected retention data under varying chromatographic conditions to build predictive regression models. QSRR approaches use molecular descriptors to capture structural and physicochemical properties of analytes and apply statistical or machine learning methods to predict RTs. QSRR approaches have been explored in several studies. For instance, Bouwmeester et al.^7^ empirically evaluated a range of machine-learning algorithms for RT prediction using molecular descriptors. Domingo-Almenara et al.^8^ introduced the METLIN small molecule retention time (SMRT) data set and developed a deep learning model for retention time prediction. Bonini et al.^9^ introduced the R package Retip, enabling RT prediction based on learned models. The RT-Pred web server^10^ similarly lets users train customized RT predictors for their own chromatographic methods. Osipenko et al.^11^ trained machine learning models to predict RTs from molecular representations (e.g., fingerprints and molecular descriptors). Liapikos et al.^12^ compared several regression algorithms, including support vector regression, to construct predictive models. Zhang et al.^13^ likewise included a QSRR model based on molecular descriptors to predict RTs within a chromatographic method. Kang et al.^14^ trained a deep graph convolutional network model for RT prediction (DeepGCN-RT).

In addition to predicting RT directly from the molecular structure, several methods focus on transferring RT between chromatographic setups using projection models. In this work, we refer to the corresponding task as the model adjustment problem: adapting an existing RT-prediction model to slightly modified chromatographic conditions using a small set of metabolites remeasured under the new setup. Stanstrup et al.^15^ implemented the PredRet system to transfer RT information between chromatographic setups using projection models learned from compounds measured on both setups. The projection models are monotonically constrained generalized additive models (GAMs). Osipenko et al.^11^ have proposed a two-step approach where RTs are first predicted from molecular descriptors in a source setup and then projected to the target setup using a piecewise linear transfer function fit on a small set of compounds measured on both setups. Zhang et al.^13^ likewise rely on projecting RTs between chromatographic methods but model the projection nonlinearly by Gaussian process regression and introduce a post–projection calibration step using external calibrants to reduce systematic setup effects. More recently, Kretschmer et al.^16^ instead predict a chromatographic-condition-aware retention-order index and map it to retention time, transferring to new setups without training data measured on them. Recent advances in QSRR methodology focus on learned molecular representations and cross-system chromatographic transferability. For example, Mazraedoost et al.^17^ proposed a hybrid transformer–Long Short-Term Memory (LSTM) model based on Simplified Molecular Input Line Entry System (SMILES) representations and evaluated it on the SMRT data set as well as on multiple PredRet data sets. Cheng et al.^18^ introduced a graph-based atom–bond colearning framework and evaluated it using the SMRT data set, followed by fine-tuning on external chromatographic systems. Similarly, Kang et al.^14^ explored fine-tuning the DeepGCN-RT model on external data sets via transfer learning and Kumari et al.^19^ examined transfer learning for multitarget QSRR modeling under heterogeneous RPLC conditions. RepoRT^20^ provides a comprehensive RT repository with chromatographic metadata relevant to cross-system prediction.

Here we present FastRet, an RT prediction framework that is available both as an R package and through a web-based graphical user interface (GUI). While recent studies focused on improving molecular representations and transfer-learning techniques, FastRet is designed around practical metabolomics workflows. The framework supports three related tasks: First, training RT prediction models from scratch using a large set of compounds and their molecular descriptors. Second, training new RT prediction models for modified chromatographic conditions from scratch using a limited set of compounds. For this, the most informative ones will be selected for remeasurement. Third, adjusting predicted RTs to slightly modified chromatographic conditions using only a small set of remeasured metabolites. In the following, we describe these methods and evaluate them on RP, RP-AXMM, and HILIC data sets, including an external HILIC data set, an independent adjustment-validation experiment and additionally 14 external data sets.

## Methods

### Chemicals

Metabolite standards, formic acid and ammonium formate were purchased from Sigma-Aldrich (Taufkirchen, Germany) and HPLC–MS grade acetonitrile (ACN) for LC–MS analysis from VWR (Vienna, Austria). Purified water was obtained from a PURELAB Plus system (ELGA, LabWater, Celle, Germany).

### Instrumentation

A Thermo Scientific Dionex Ultimate 3000 UHPLC system (Idstein, Germany) was interfaced with a Maxis Impact QTOF–MS (Bruker Daltonics, Bremen, Germany) using an ESI source. Three types of columns were used: a HILIC, a RP, and a RP-AXMM column. In the Supporting Information, details are provided on the columns (Table S1), eluents (Table S2), and gradient conditions (Table S3). Electrospray ionization (ESI) was performed in positive and negative ionization modes using the following settings to operate the source and the mass spectrometer: drying gas (nitrogen) temperature of 220 °C and flow rate of 10 L/min; nebulizer gas pressure (nitrogen): 2.6 bar; end plate offset: 500 V; capillary voltage: 4500 V; mass range: 50–1000 *m*/*z*; acquisition rate: 2 spectra/s. An external calibration of the mass spectrometer was employed before measurements using a sodium formate cluster solution (10 mM sodium formate in 50:50 v/v water/isopropanol). For internal recalibration, each run involved an injection of the sodium formate cluster solution using a six-port valve. Mass spectral resolutions of *R* = 22000 (for *m*/*z* 90.977) were obtained.

### Data Sets

We analyzed a total of 18 data sets. Data sets 1–4 were obtained by measuring the metabolite standards on LC columns with different separation mechanisms: reversed-phase chromatography (RP), hydrophilic interaction liquid chromatography (HILIC) and reversed-phase–anion-exchange mixed-mode chromatography (RP-AXMM). Because not all metabolites were detectable under each chromatographic condition, data set sizes vary. The coverage of different chemical compound classes by these data sets is shown in Figure 1, using RP as an exemplary data set. Data set 5 was obtained from the R package Retip^9^ published by Bonini et al. Data sets 1–4 and 6–18 are available as part of the FastRet package or can be downloaded directly as xlsx file from https://github.com/spang-lab/FastRet/releases/download/example-data/Measurements_v10P.xlsx. Data sets 1–5 were used to estimate performance of model training and model adjustment by splitting data sets into (paired) training and test sets as described in the Estimating Performance section. Data sets 6–11 consist of a subset of 25 metabolites (Supporting Information Table S4), selected from an early version of Data set 1 using the SM1 selective measuring strategy (Selective Measuring for Training of New or Adjustment of Existing Models section), and remeasured under different chromatographic conditions. Data sets 12–18 comprise a new set of 22 metabolites (Supporting Information Table S5), measured under the same conditions as Data sets 1 and 6–11. Data sets 6–18 were used to estimate the performance of model adjustment using data from independent experiments. In running text, data set names are written with hyphens (e.g., HILIC-Retip and RP-AXMM), whereas code-style identifiers use underscores (e.g., HILIC_Retip and RP_AXMM). All data sets are summarized in Table 1. Their overlap is visualized in Figure S1.

**Figure 1:**
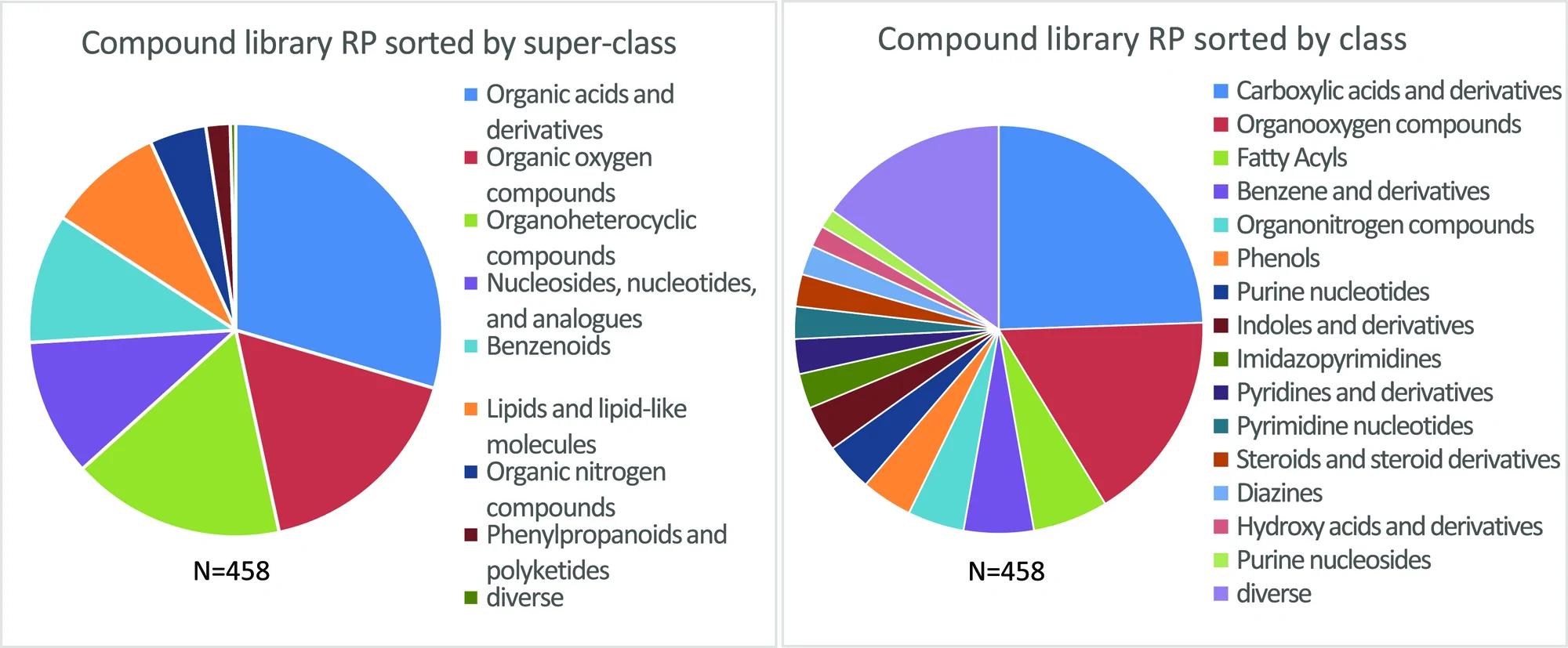
Pie charts showing the compound superclass (left) and class (right), as reported in their corresponding HMDB^21^ entries. The exemplary coverage shown is based on the RP data set, with RP-AXMM and in-house HILIC data sets showing similar compound-class coverage.

**Table 1:** Summary of Data Set Used in This Study^a^

Nr	ID	No	Column	Phase A	Phase B	Gradient	FR	Temp
1	RP	458	HSS-T3	FA100-W	FA100-A	Norm	0.3	35
2	RPB	458	HSS-T3	FA100-W	FA100-A	NormB	0.3	35
3	RP_AXMM	436	BEH-AX	FA100-AF10-W	FA100-AF10-WA10	AX	0.3	35
4	HILIC	388	BEH-Amide	FA125-AF10-W	FA125-AF10-WA5	HILIC	0.4	45
5	HILIC_Retip^9^	970	BEH-Amide	FA125-AF10-W	FA125-AF10-WA5	Retip	0.4	45
6	RP_Flat	25	HSS-T3	FA100-W	FA100-A	Flat	0.3	35
7	RP_FR25_Flat	25	HSS-T3	FA100-W	FA100-A	Flat	0.25	35
8	RP_Steep	25	HSS-T3	FA100-W	FA100-A	Steep	0.3	35
9	RP_T25_Flat	25	HSS-T3	FA100-W	FA100-A	Flat	0.3	25
10	RP_T25_FR25_Flat	25	HSS-T3	FA100-W	FA100-A	Flat	0.25	25
11	RP_T25_FR25_Steep	25	HSS-T3	FA100-W	FA100-A	Steep	0.25	25
12	RP_Val	22	HSS-T3	FA100-W	FA100-A	Norm	0.3	35
13	RP_Val_Flat	22	HSS-T3	FA100-W	FA100-A	Flat	0.3	35
14	RP_Val_FR25_Flat	22	HSS-T3	FA100-W	FA100-A	Flat	0.25	35
15	RP_Val_Steep	22	HSS-T3	FA100-W	FA100-A	Steep	0.3	35
16	RP_Val_T25_Flat	22	HSS-T3	FA100-W	FA100-A	Flat	0.3	25
17	RP_Val_T25_FR25_Flat	22	HSS-T3	FA100-W	FA100-A	Flat	0.25	25
18	RP_Val_T25_FR25_Steep	22	HSS-T3	FA100-W	FA100-A	Steep	0.25	25

a Columns show: data set number, data set ID, number of compounds (No), column ID (Table S1), eluent IDs (Table S2), gradient ID (Table S3), flow rate (mL/min), and column temperature (°C).

### Problem Definition

FastRet implements three related tasks: (1) training a RT model from scratch, (2) selecting an informative subset of compounds to measure, and (3) adjusting an existing model to modified chromatographic conditions. Throughout, a compound *i* is represented by a feature vector $x_{i}\in R^{p}$ containing *p* chemical descriptors introduced in the Chemical Descriptor Computation section. For a given chromatographic setup, the corresponding data set is *D* = {(*x*_*i*_, *y*_*i*_)}_*i* = 1_^*n*^ where $y_{i}\in R_{>0}$ denotes the RT of compound *i* measured with that setup.

When training from scratch, FastRet takes a data set *D* and learns a predictor $f:R^{p}\to R_{>0}$ that maps the descriptors of a compound to its RT, *ŷ* = *f*(*x*). The predictor is fitted by minimizing a regularized loss on *D* using either Lasso or BRT. The resulting model can then predict RTs for unseen compounds measured under the same chromatographic setup.

In selective measuring, the input is a pool of *N* candidate compounds with known descriptors, of which only *k* compounds should be experimentally measured. The aim is to identify the subset of size *k* that is expected to be most informative for model training. FastRet achieves this by clustering compounds in a ridge-weighted descriptor space and selecting the *k* cluster medoids.

Model adjustment is used when chromatographic conditions change and a RT prediction model for the original setup is already available. Rather than training a new model from scratch, FastRet uses a small set of compounds measured under both chromatographic conditions to learn how RTs change between setups. Model adjustment starts from the base model *f*_base_ trained for the original setup and a small set of compounds remeasured under the modified chromatographic conditions. For each remeasured compound we know the chemical descriptors *x*, the original-setup RT *y*, and the new RT *y*′ under the modified conditions. Based on these paired measurements, FastRet learns an adjustment model *g* that predicts the modified-setup RT from the descriptors and the original-setup RT, *ŷ*′ = *g*(*x, y*). The linear adjustment model uses only the original RT, *g*(*x, y*) = α + *βy*, corresponding to a direct RT-to-RT projection. In contrast, the Lasso and BRT adjustment models use both descriptors and the original RT, allowing compound-specific adjustment effects to be learned. For a new compound that has not been measured under the original conditions, RT is first predicted with the base model, *y* ≈ *f*_base_(*x*), and then given to the adjusted FastRet model *x* → *g*(*x*, *f*_base_(*x*)). The aim of model adjustment is to improve prediction accuracy under modified chromatographic conditions while requiring only a limited number of new RT measurements.

Both training from scratch and model adjustment require a set of compounds to be measured under the new chromatographic conditions. Selective measuring minimizes this experimental effort by recommending which compounds to remeasure. Figure 2 summarizes the three main FastRet workflows: (1) training a model from scratch, (2) selecting metabolites for remeasurement under limited measurement budgets, and (3) adjusting an existing model to modified chromatographic conditions.

**Figure 2:**
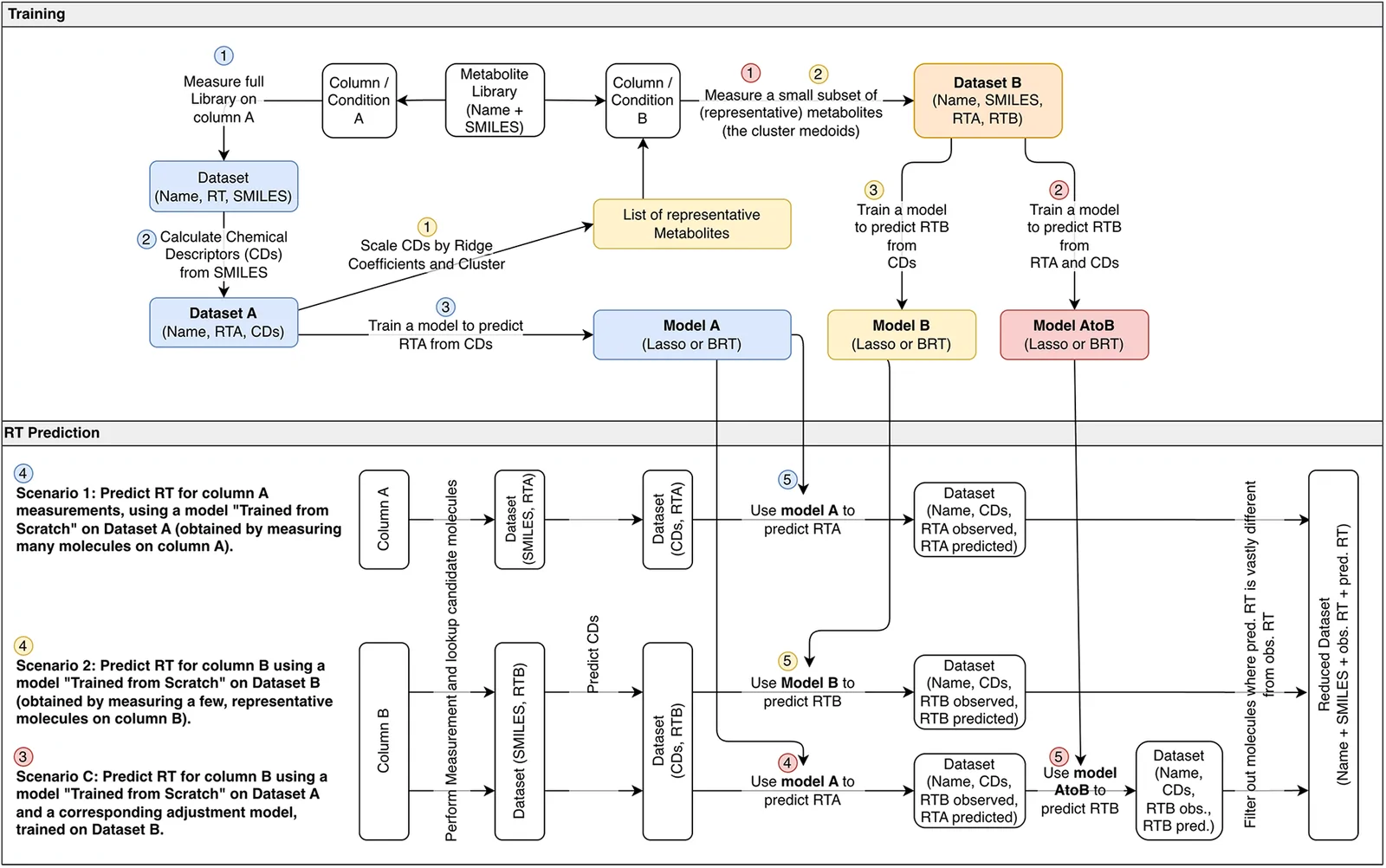
Overview of the three main FastRet workflows. Data sets and models related to Train-from-Scratch are shown in blue, those related to Selective-Measuring are shown in yellow and those related to Model-Adjustment are shown in red. Data set B is shown in orange, indicating that it is used in both the Selective-Measuring and Model-Adjustment workflows. The order in which the individual steps are executed is indicated by the numbers in the circles. Both Selective-Measuring and Model-Adjustment can only be performed if a base model is present, as they both depend on inputs from the base model. (BRT: Boosted Regression Trees; CD: chemical descriptors; LASSO: linear regression with Least Absolute Shrinkage and Selection Operator; RTA: retention time on column A and/or with condition A; RTB: retention time on column B and/or with condition B).

### Chemical Descriptor Computation

In FastRet, the feature vectors are chemical descriptors computed from molecular structures provided as SMILES strings (Simplified Molecular Input Line Entry System). Chemical descriptors are numeric summaries of molecular structure, including constitutional, topological, and physicochemical properties. We compute them with the rcdk package,^22,23^ an R wrapper around the Chemistry Development Kit (CDK).^24^ We use 241 descriptors. This set was obtained by listing all descriptors available through rcdk with version 2.3 of the underlying rcdklibs package and excluding any descriptor that produced a warning or error for at least one compound in our library. For example, for glucose with SMILES C­([C@@H]­1­[C@H]­([C@@H]­([C@H]­(C­(O1)­O)­O)­O)­O)­O, rcdk returns descriptors such as molecular weight (MW = 180.16), topological polar surface area (TopoPSA = 110.38), and number of hydrogen-bond donor sites (nHBDon = 5). The 241 descriptors span constitutional, topological, electrotopological and physicochemical families and are summarized in Table S6.

To keep preprocessing fast in repeated use, FastRet caches descriptor values by SMILES string. Repeated requests for already known compounds therefore reduce to cache lookups, and only previously unseen SMILES are evaluated with rcdk. Descriptor computation for new compounds can be parallelized across workers. The chemical descriptor computation is summarized in Supporting Information Listing S1.

Numerous other packages compute molecular descriptors—among them PaDEL,^25^ Mordred,^26^ RDKit,^27^ and the commercial Dragon/alvaDesc^28^—but nearly all require a separate Python or Java toolchain. We chose rcdk because it is, to our knowledge, the only descriptor engine callable directly from within R. The one apparent R-native alternative, Rcpi,^29^ itself delegates to rcdk. Although CDK is written in Java, this is fully abstracted by rcdk, so FastRet stays a single R dependency that is easy to install and maintain.

### Training Models from Scratch

FastRet allows training of RT-prediction models from a list of compounds, identified by their SMILES string, and their measured RTs. FastRet trains a single *global* model rather than *local* models restricted to compounds structurally similar to a target, because in untargeted metabolomics the analytes are unknown and chemically diverse, precluding a per-target training set. Since RT depends on the chromatographic column and conditions, each model is specific for a certain column and/or experimental setup. As soon as either the column or the conditions change, a new model must be trained or an existing model must be adjusted (see Adjustment of Existing Models section).

Depending on the chemistry of the column and the chromatographic conditions either a linear or a nonlinear prediction model may be optimal. Therefore, FastRet offers both possibilities by using either linear regression employing LASSO^30^ (via the glmnet package^31^) or for a nonlinear model Boosted Regression Trees^32^ (via the xgboost package^33^). For Lasso, FastRet uses glmnet with internal cross-validation to select the penalty parameter λ and then refits the model at the selected value. For the data set sizes considered here, this step typically finishes in below one second on a standard desktop computer. For BRT, FastRet uses xgboost with default parameters except for nrounds, which is optimized by internal cross-validation with early stopping. This stops training as soon as the cross-validated performance no longer improves, avoiding unnecessary boosting iterations. A more detailed description of the two model families is given in Supporting Information Notes S1 (Lasso) and S2 (Boosted Regression Trees). The input features for model training are the chemical descriptors described in the Chemical Descriptor Computation section. Both model types are well suited for settings with more features than samples and therefore require no separate descriptor preselection or dimensionality reduction. The Lasso $l_{1}$ penalty regularizes the fit by shrinking most coefficients to zero, while boosted regression trees weight features by their contribution to the split gain and are robust to correlated and uninformative descriptors. By default, FastRet also returns a cross-validated performance estimate. This outer performance estimation wraps model fits that themselves use internal cross-validation for hyperparameter selection, yielding a nested cross-validation procedure. The outer folds can be trained in parallel via the nw (number of workers) parameter. Together with descriptor caching, these implementation choices limit typical model-training runtimes to a few seconds. The training procedure is summarized in Supporting Information Listing S2.

### Selective Measuring for Training of New or Adjustment of Existing Models

Each metabolite that needs to be measured to achieve satisfactory model performance poses an expenditure of time and resources. Thus, there is a trade-off between measurement effort and predictive performance. We propose a selective measuring (SM) strategy that recommends a subset of *k* ≤ *N* metabolites from a base data set consisting of *N* metabolites, measured under similar chromatographic conditions. The idea is to cluster metabolites in a feature space composed of chemical descriptors and RTs, and to remeasure one representative per cluster. We use a two-step procedure. To emphasize variables most informative for RT prediction, each feature is first scaled by its coefficient from a Ridge regression,^31^ modeling RT as a function of chemical descriptors. We use Ridge instead of Lasso in this context, because it only shrinks features that are less informative in the base data set, rather than eliminating them completely. This accounts for the fact that the set of informative features may change under different chromatographic conditions. Next, we cluster metabolites based on the scaled chemical descriptors and RTs to select informative metabolites. For clustering, we apply the *k*-medoids algorithm via the PAM (Partitioning Around Medoids)^34^ implementation from the R package cluster, using Euclidean distances in the scaled feature space. Unlike *k*-means, *k*-medoids selects actual data points as cluster centers (medoids), which directly identifies representative metabolites for selective remeasurement. To control the influence of RT during clustering, we scale the standardized RT feature with the parameter rt_coef, i.e., rt_coef = 0 drops RT (SM0), and rt_coef = 1 keeps it unchanged (SM1). The max option scales RT by multiplying it with the largest absolute Ridge coefficient (SMmax), whereas the inf option zeroes all other features so RT is “infinitely” more important than any other feature (SMinf). The method yields a selection of metabolites that spans the observed RT range and/or the relevant descriptor variation, which are both important for model training. The algorithm is described in Supporting Information Listing S3.

### Adjustment of Existing Models

If chromatographic conditions change, RTs shift accordingly. However, as long as this shift is minor, RTs measured under the original conditions remain informative for the modified ones. Previous studies by Stanstrup et al.,^15^ Osipenko et al.^11^ and Zhang et al.^13^ exploit this with projection models that map the RT under the original conditions (RT-base) directly to the RT under the modified conditions (RT-adjusted). Hence, most models can only be applied to compounds whose RT-base has already been measured.

FastRet extends this idea in two ways. First, its adjustment model predicts RT-adjusted not from RT-base alone, but from RT-base together with the compound’s chemical descriptors. We therefore call these adjustment models rather than projection models. Second, while the adjustment model is still trained on compounds measured under both conditions, for prediction it operates on a predicted rather than a measured RT-base. Specifically, the base model first predicts RT-base from the chemical descriptors, and the adjustment model then predicts RT-adjusted from that prediction and the same descriptors. This enables FastRet to adjust predictions even for compounds that were not measured under the original conditions.

For both Lasso and BRT, the fitting routine and internal hyperparameter optimization are otherwise unchanged. Internal cross-validation is used for penalty or round selection, and BRT fitting again uses early stopping to avoid unnecessary boosting rounds. By default, FastRet also reports a cross-validated performance estimate for the adjustment step. The algorithm for fitting an adjusted FastRet model is described in Supporting Information Listing S4.

### Predicting RTs

Predictions are obtained by applying the base model trained from scratch to new data after ensuring required descriptors are available. If predictors are missing, they are computed from SMILES strings. Chemical descriptors cannot be computed completely for every molecular structure, resulting in missing values. These are imputed as the mean of the corresponding predictor observed in the training data. We consider mean imputation adequate here, since only a very small fraction of descriptor values is affected. Across all 1137 compounds, only 0.43% of the 241 descriptor values are undefined, and nearly all belong to a single descriptor that requires a 3D geometry FastRet does not generate. Being undefined for every compound, it is always dropped during training and never imputed. Only 35 values (0.011%), across 13 descriptors and 4 compounds, are actually imputed. Predicted RTs are clipped to a plausible range derived from the training data. The bounds are computed from a log-normal fit to observed RTs, then adjusted so that the lower bound lies between 0 and the observed minimum, and the upper bound is at least the observed maximum. The exact procedure is given in Supporting Information Listing S5. If an adjustment model exists, predictions are obtained by first predicting with the base model, then using those predictions as input to the adjustment model, which also considers chemical descriptors. The adjustment model predictions are then clipped again based on the training data of the adjustment model. The prediction procedure is summarized in Supporting Information Listing S6.

### Estimating Performance

Performance of adjusted or newly trained models is estimated using the procedure described in Supporting Information Listing S7. The procedure builds training and test splits and evaluates predictions under each setting.

As can be seen in Listing S7, the procedure takes a total of 6 parameters, defining the data sets used for training and testing, the number of compounds used for training, the selection method for choosing the training measurements, the model types for training the base and final models, and the random seed for reproducibility. If sel_meth is Random, the seed determines which compounds are drawn for training. For selective-measuring methods, it affects the cluster initialization used to select the representative compounds. During model fitting, the seed also affects the internal cross-validation folds used for hyperparameter tuning.

We use dedicated training and test data sets instead of cross-validation, because adjusted-model performance depends on predictions from both the base model and the adjustment model. Therefore, to obtain unbiased estimates, test-set compounds must not appear in either the base or the training set. Cross-validation would therefore require paired folds of both base and training data, increasing complexity and reducing interpretability.

Constructing valid base/training/test splits is further constrained by the selective-measuring evaluation. Every compound in the base set (i.e., the data set used for training the base model and as the pool for selective measuring) must be available in the training set, because any base compound may be recommended for remeasurement when simulating different subset sizes. To evaluate model adjustment, every training compound must also be present in the base set. Together, these constraints require base and training data sets to contain identical compound sets for comprehensive performance estimation. The construction of these subsets is outlined in Supporting Information Listing S8. The resulting subsets are visualized in Figure 3. The data set combinations used for performance estimation are listed in Table 2.

**Figure 3:**
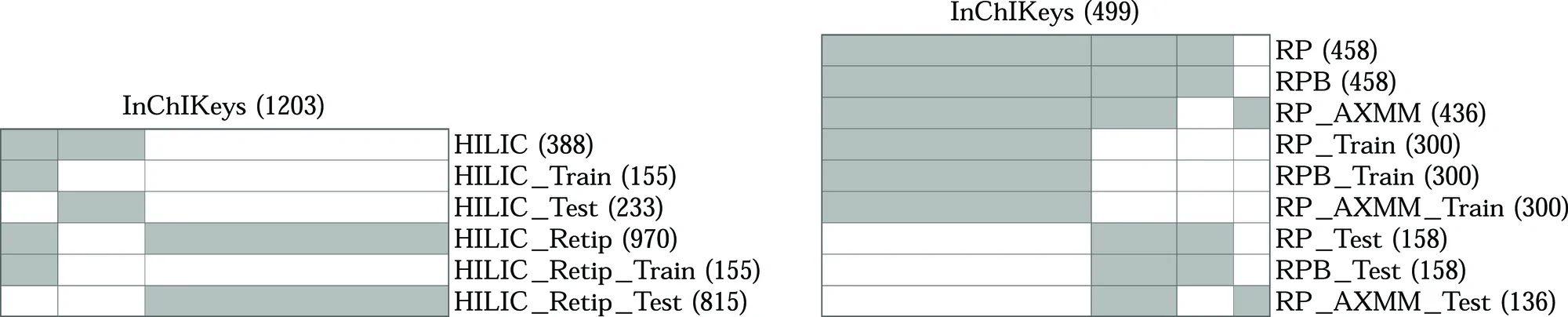
Heatmaps visualizing the presence of compounds across training-/test subsets. Left: HILIC and HILIC-Retip data sets. Right: RP, RPB and RP-AXMM data sets. Each row represents a data set, columns represent sets of compounds, identified by InChIKeys (a fixed-length hash of the IUPAC International Chemical Identifier that uniquely represents a molecular structure). The *x*-axis spans all distinct compounds across all data sets combined. The tick marks per compound have been omitted to improve readability. A filled cell indicates that the compound set is present in the respective data set. Vertical lines separate groups of compounds by their data set membership, e.g., compounds shared by all data sets versus those unique to one. Data set IDs are given on the right. The number of compounds in a data set is given in brackets.

**Table 2:** Data Set Combinations Used in This Study^a^

Nr	Base	Train	Test
1	RPB_Train	RP_Train	RP_Test
2	RP_Train	RP_AXMM_Train	RP_AXMM_Test
3	HILIC_Retip_Train	HILIC_Train	HILIC_Test
4	RP	RP_Flat	RP_Val_Flat
5	RP	RP_FR25_Flat	RP_Val_FR25_Flat
6	RP	RP_Steep	RP_Val_Steep
7	RP	RP_T25_Flat	RP_Val_T25_Flat
8	RP	RP_T25_FR25_Flat	RP_Val_T25_FR25_Flat
9	RP	RP_T25_FR25_Steep	RP_Val_T25_FR25_Steep

a Data set IDs correspond to Table 1. Columns “Base”, “Train” and “Test” indicate the role of each data set during performance estimation.

## Results and Discussion

### From Scratch Performance

We compared the performance of models trained from scratch as described in the Estimating Performance section using the data sets RP, RP-AXMM and HILIC. Subsets were drawn from their training sets (RP-Train, RP-AXMM-Train, HILIC-Train) randomly without replacement and the performance was evaluated using the respective test set. Each random experiment was repeated at least 10 times, and the coefficient of determination (*R* ^2^) and the mean absolute error (MAE) in minutes were calculated. *R* ^2^ and MAE for different training subset sizes are visualized in Figure 4. When trained on smaller training subsets (up to approximately 50 metabolites), BRTs and Lasso showed similar performance with respect to their *R* ^2^ and MAE. Both for the RP and the HILIC data set a plateau was reached for BRT performance with a training size of approximately 100 metabolites. However, for RP-AXMM, the performance of both BRT and Lasso models continued to improve with increasing subset size. Using the full training sets resulted in the best performance for RP (MAE of 0.65 for BRT and 0.95 for Lasso) followed by HILIC (MAE of 1.00 for BRT and 1.28 for Lasso). The worst performance was observed for the RP-AXMM data set with an MAE of 2.29 for BRT and 2.95 for Lasso. Distributions of *R* ^2^ at *n* = 25 are shown in Figure 5. The median difference in *R* ^2^ between BRT and Lasso was small for *n* = 25: −0.01 for RP, −0.06 for HILIC and 0.01 for RP-AXMM. Despite these small differences in median, BRT is the more stable choice, with fewer negative outliers than Lasso (Figure 5), and achieves a significantly higher mean *R* ^2^ on all three data sets (Holm-adjusted paired *t*-tests over 1000 shared seeds, all *p* < 0.001; mean differences and 95% confidence intervals in Supporting Information Table S7). This advantage grows with training-set size. When trained on the full training sets, BRT consistently outperformed Lasso, with a median difference in *R* ^2^ of 0.11 for RP, 0.07 for HILIC and 0.26 for RP-AXMM. Observed training times on the RP data set measured on the MacBook Pro (see Supporting Information Table S8) were below 1 s without outer cross-validation and about 2.6–2.8 s with default cross-validated performance estimation (Supporting Information Table S9).

**Figure 4:**
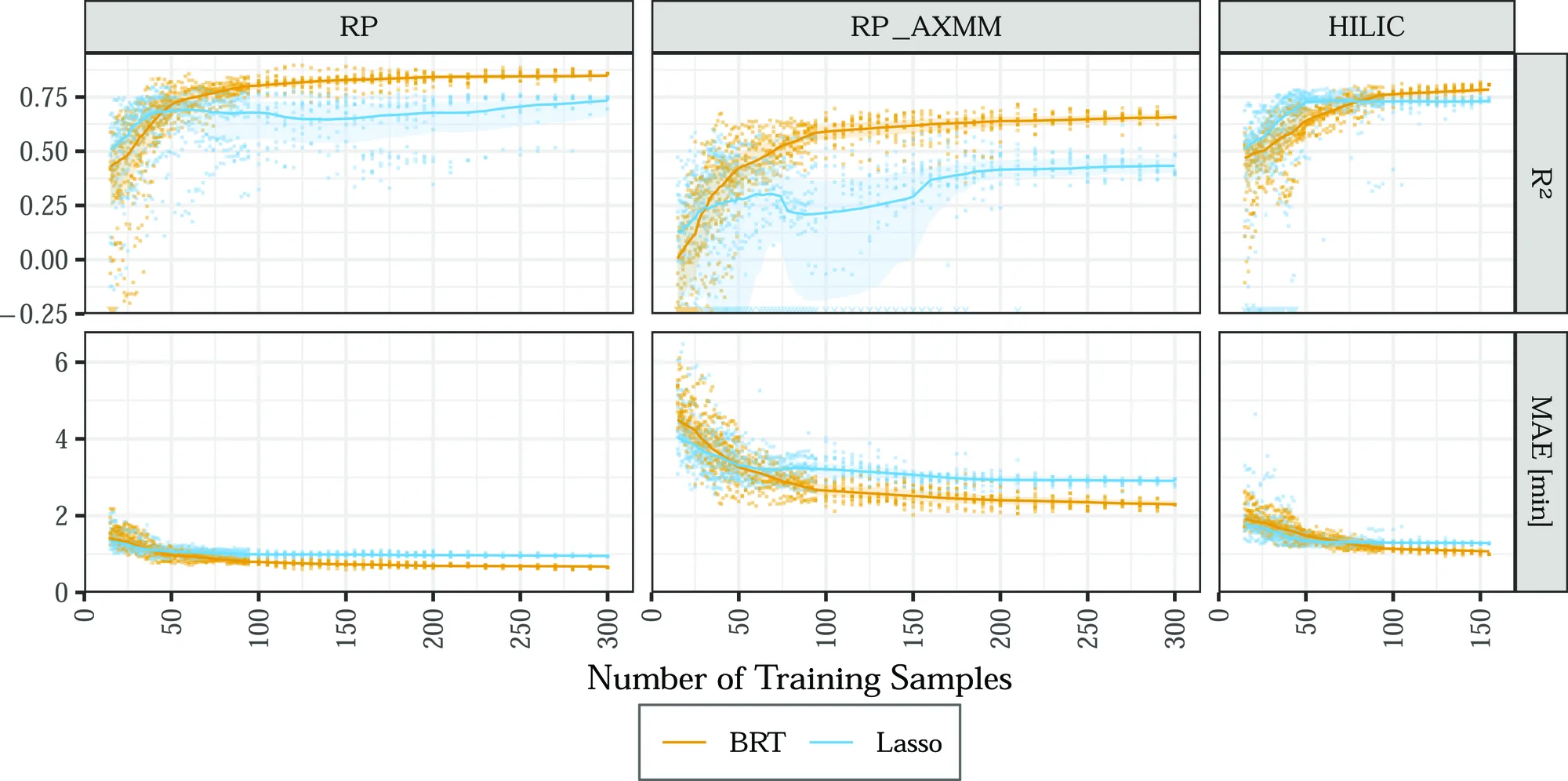
From-Scratch-Performance across training subset sizes for RP, RP-AXMM and HILIC data sets. Top panel shows *R* ^2^ (values below −0.25 are plotted as crosses at −0.25). Bottom panel shows MAE. Each point shows one random subset drawn from the training set. For each subset size, 10 repetitions were performed using different random seeds. Solid lines and shaded bands indicate the moving-average smoothed median and interquartile range (window size = 21) across repetitions. Median and interquartile range calculations use the true *R* ^2^ values. Line colors distinguish model types (BRT and Lasso).

**Figure 5:**
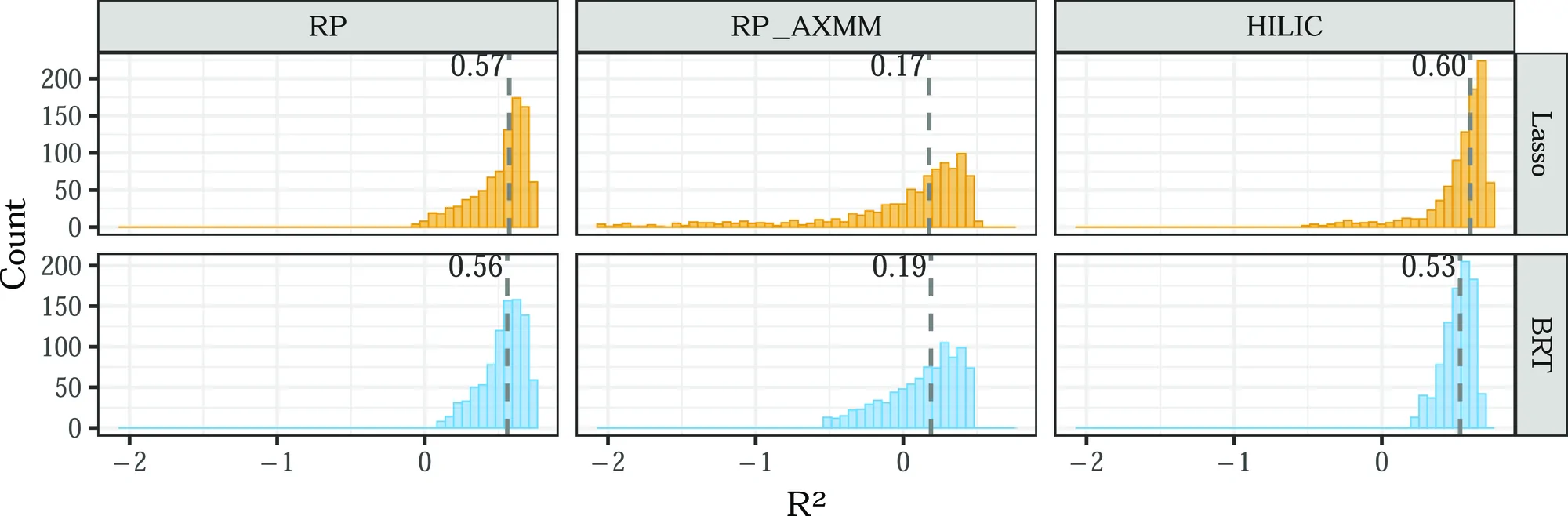
*R* ^2^ histograms for From-Scratch-Performance at *n* = 25 for Lasso (row 1) and BRT (row 2) models. Training was repeated 1000 times by randomly drawing subsets of 25 metabolites each time using a different seed. A fixed test set is used. The extreme 5% on both ends are hidden, showing only the middle 90% of the data to improve readability. The medians use the full data, including omitted 5% tails, and are visualized as dashed vertical lines.

Despite the 241 input descriptors, the fitted models were sparse: the from-scratch Lasso models retained only 15–41 descriptors with nonzero coefficients (e.g., 18 for RP), and gradient boosting concentrated importance on a comparably small set, dominated by lipophilicity indices (XLogP, ALogP). Descriptor signatures for all models are shown in Supporting Information Figure S2. This indicates that the models’ integrated feature selection effectively controls the high descriptor-to-sample ratio.

### Selective Measuring

Instead of a brute-force approach that trains prediction models from scratch using large data sets, we developed a strategy to prioritize compounds that are most informative for RT prediction. Here, we analyze how well the selection of a restricted set of compounds works for RT prediction. First, a Ridge regression model is trained to predict RT from chemical descriptors, and the resulting coefficients are used to weight the descriptors, emphasizing features relevant for RT prediction. Metabolites are then clustered using the weighted descriptors and RT, and one representative from each cluster is remeasured. We evaluated our selective measuring approach on data set combinations 1–3 (RPB/RP, HILIC-Retip/HILIC and RP/RP-AXMM). For each combination, we selected *k* compounds from the base data set based on the clustering approach, where *k* also represents the number of selected cluster centers, where the medoid compound of each cluster was selected for the training set. We restricted our training data set to these *k* compounds, trained on that training subset, and evaluated on the test data set. For example, in the case of RP chromatography, we used the RPB data set as the base to select the training compounds. The models were trained using the RT from the RP data set (training split), which was acquired under slightly different chromatographic conditions compared to RPB. While the RP data sets were all generated in-house, HILIC preselection relies on literature data (HILIC-Retip), and compound selection for RP-AXMM is based on RP data that do not account for electrostatic interactions. The performance of the trained models was evaluated on the test split of the respective data set (RP-Test, RP-AXMM-Test, HILIC-Test). The *R* ^2^ values for BRT models are visualized in Figure 6, together with the results from drawing random subsets of size *k*. MAE results for BRT models are given in the Supporting Information (Figure S3). Additional *R* ^2^ and MAE results for Lasso models are also provided in the Supporting Information (Figures S4 and S5). The performance distribution for n_train = 25 is visualized in Figure 7. The best overall strategy for training from scratch with a limited set of only 25 compounds uses the standardized RT without further scaling for clustering (SM1). Over 1000 random experiments, SM1 improved the median *R* ^2^ over random selection by 0.06 (RP), 0.05 (HILIC) and 0.31 (RP-AXMM), and this gain is statistically significant on all three data sets (Holm-adjusted paired *t*-tests over 1000 shared seeds, all *p* < 0.001); the mean differences and 95% confidence intervals are given in Supporting Information Table S7. A similar performance was obtained with SMmax that scales the standardized RT with the largest absolute Ridge coefficient for clustering. Relying only on the standardized RT for clustering (SMinf) yielded the best performance for the RP data. This is not surprising as the chromatographic conditions used to generate the RPB data set differed only in gradient starting conditions (0% eluent B for RTB versus 3% eluent B for RT). Importantly, *R* ^2^ distributions are much narrower for all selective measuring variants compared to random selection, which holds for all three columns. Interestingly, preselecting a small set of metabolites based on the RP-Train data set to train an RP-AXMM model outperforms random compound selection for training the RP-AXMM model in particular for the selective measuring variants SMmax, SM1, and SMinf. As shown in the following section, SMmax provides the most robust performance when selective measuring is combined with model adjustment, making it the recommended strategy for practical applications.

**Figure 6:**
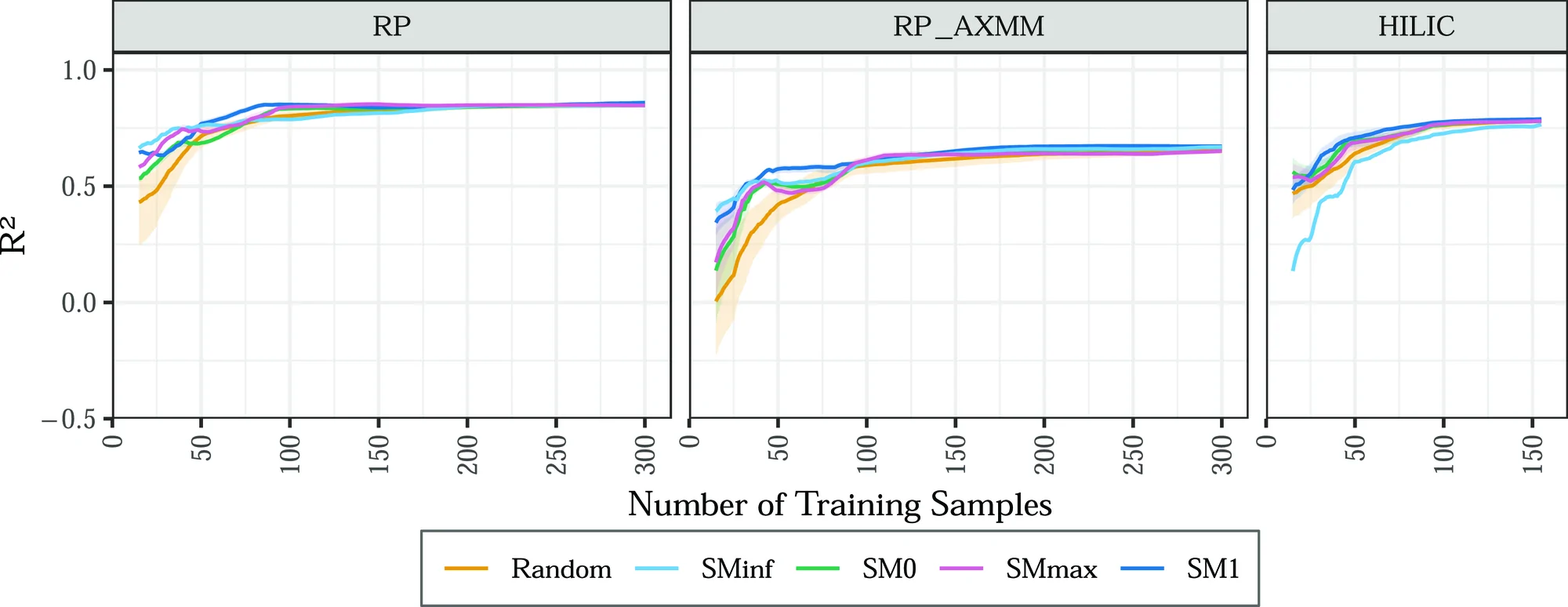
Selective-Measuring-Performance for BRT models (*R* ^2^) across training subset sizes for RP, RP-AXMM and HILIC data sets. Ten repetitions were performed using different random seeds for each parameter combination (selection strategy and training subset size). Plots compare different selection strategies (lines) across training subset sizes (*x*-axis) and show *R* ^2^ performance (*y*-axis). The lines show: Random selection, and four selective measuring variants (SMinf, SM0, SMmax, SM1) that differ in how they use and scale retention times (RT) for clustering (see Methods section for details). Solid lines represent the median performance across the 10 runs, and shaded areas show the interquartile range (middle 50%). Both lines and shaded areas are smoothed using a moving average (window size 21) for better readability.

**Figure 7:**
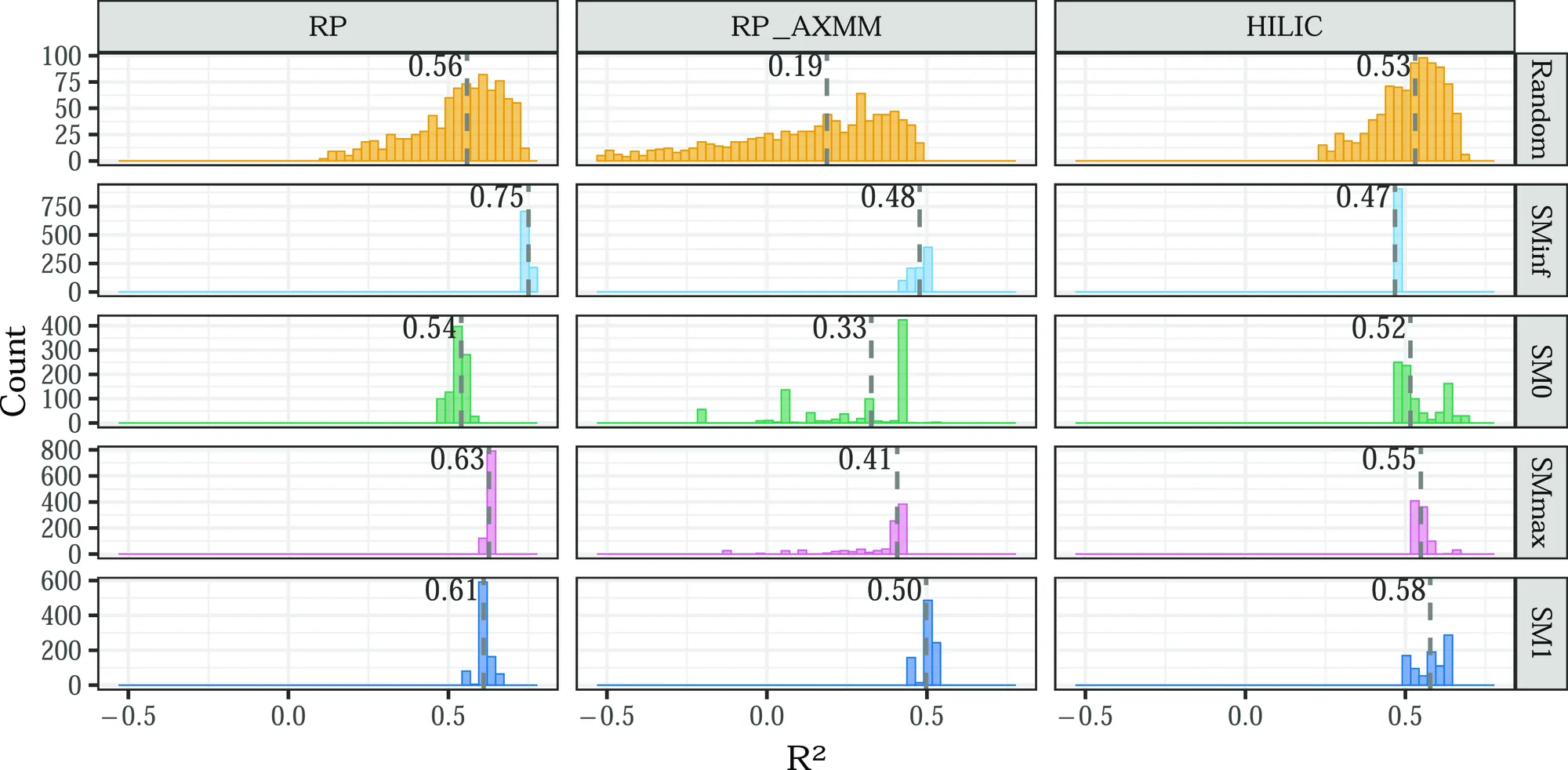
Selective-Measuring-Performance as *R* ^2^ distributions for BRT models at *n* = 25, grouped by selection method. Each selection strategy was repeated 1000 times using different random seeds. For the Random strategy, seeds determined training subset sampling. For SMinf, SM0, SMmax, and SM1, the seeds affect clustering initialization and the internal cross-validation folds used for hyperparameter tuning. Only the middle 90% of the data is shown to improve readability. The medians use the full data, including omitted 5% tails.

### Adjustment Performance

Adjustment performance was evaluated as described in the Estimating Performance section, using the same data set combinations employed in evaluating the selective measuring approach: RPB/RP, HILIC-Retip/HILIC and RP/RP-AXMM. The RT correlations across base and training data sets are shown in Supporting Information Figure S6. As expected, the best correlation was observed for the RP data set combination (*r* = 0.994), followed by the HILIC combination (*r* = 0.926). Not surprisingly, the RT correlation between RP and RP-AXMM was rather poor (*r* = 0.797), due to substantial differences in column chemistry and retention mechanisms between the reversed-phase and mixed-mode anion-exchange columns. Models were trained on the base data set. A subset of n_train compounds was selected from the training data set, either randomly or via a selective measuring strategy. An adjustment model was then fitted on that subset using a regression method (Lasso, BRT, or linear model) to learn the RT shift from the selected compounds. The observed *R* ^2^ performance obtained using a BRT base model in combination with different adjustment model types is visualized in Figure 8 for compounds selected using the SMmax selective measuring approach.

**Figure 8:**
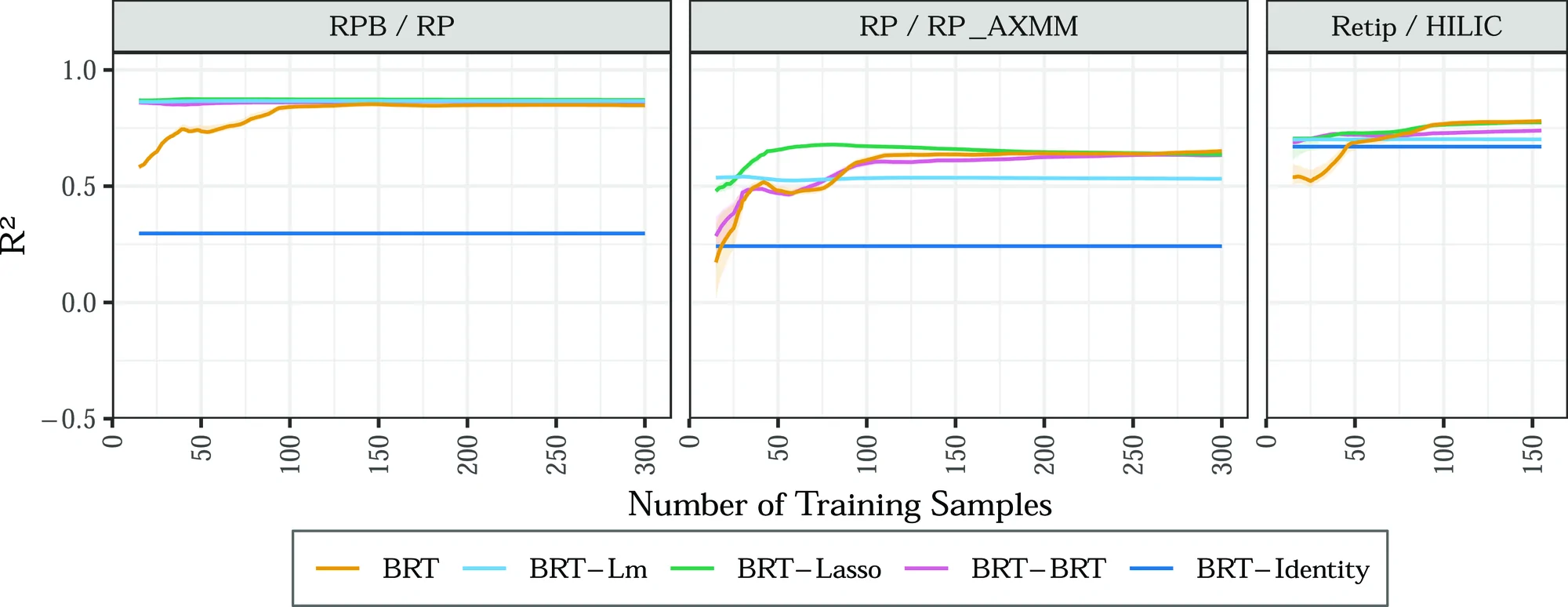
Adjustment performance (*R* ^2^) for BRT base models using SMmax across training set sizes for RPB/RP, RP/RP-AXMM and Retip/HILIC data set combinations. For each adjustment model and subset size, 10 repetitions were performed using different random seeds. The base model was always trained on the full base data set. Lines represent adjustment model types: BRT (trained from scratch), BRT–Lm (linear adjustment), BRT–Lasso (Lasso adjustment), BRT–BRT (BRT adjustment), and BRT–Identity (base model without adjustment). Solid lines show the median across the 10 repetitions. Shaded bands show the interquartile range.

Comparing the linear adjustment, which uses the base RT as its only predictor, with the Lasso and BRT adjustments, which additionally use the molecular descriptors, shows how much the descriptors contribute beyond the base RT. For the less strongly correlated combinations (most clearly RP/RP-AXMM, *r* = 0.797, but also, less markedly, HILIC-Retip/HILIC), the descriptor-based models increasingly outperform the linear adjustment as the training set grows, indicating that the descriptors carry retention-relevant information beyond the base RT. For the strongly correlated RP combination (*r* = 0.994), where the base RT almost perfectly explains the target RT, the Lasso and BRT adjustments only match the linear model and select almost no descriptors besides the base RT (Supporting Information Figure S2), confirming that their regularization adds features only when these improve on the base RT.

Among the selective measuring strategies evaluated (see Supporting Information Table S10 for a full comparison), SMmax yielded the highest and most consistent adjustment performance across all three data sets. For *n*_train = 25, the best-performing combination used a BRT base model, SMmax selective measuring strategy and Lasso adjustment model (Figure 9). This indicates that when only a small adjustment set is available, a regularized linear model such as Lasso is preferable to BRT for the adjustment step. Because the linear adjustment estimates only an intercept and a slope, it requires very few compounds for a stable fit and performs well when the relationship between base and adjusted RTs is approximately linear, as is the case for the RP data set combination. However, when the RT shift involves nonlinear or compound-specific effects, the linear model cannot compete with multivariate methods such as Lasso or BRT that use chemical descriptors as additional predictors. The latter yielded median *R* ^2^ values of 0.87 for RP/RPB, 0.70 for HILIC/HILIC-Retip and 0.54 for RP/RP-AXMM, approaching the performance of training-from-scratch on the full training sets (0.86 for RP, 0.81 for HILIC and 0.66 for RP-AXMM) using only 25 remeasured compounds. Adjusting an existing model in this way also achieves a significantly higher mean *R* ^2^ than training a model from scratch on the same 25 compounds across all three data set combinations (Holm-adjusted paired *t*-tests over the 1000 shared seeds, all *p* < 0.001; Supporting Information Table S7).

**Figure 9:**
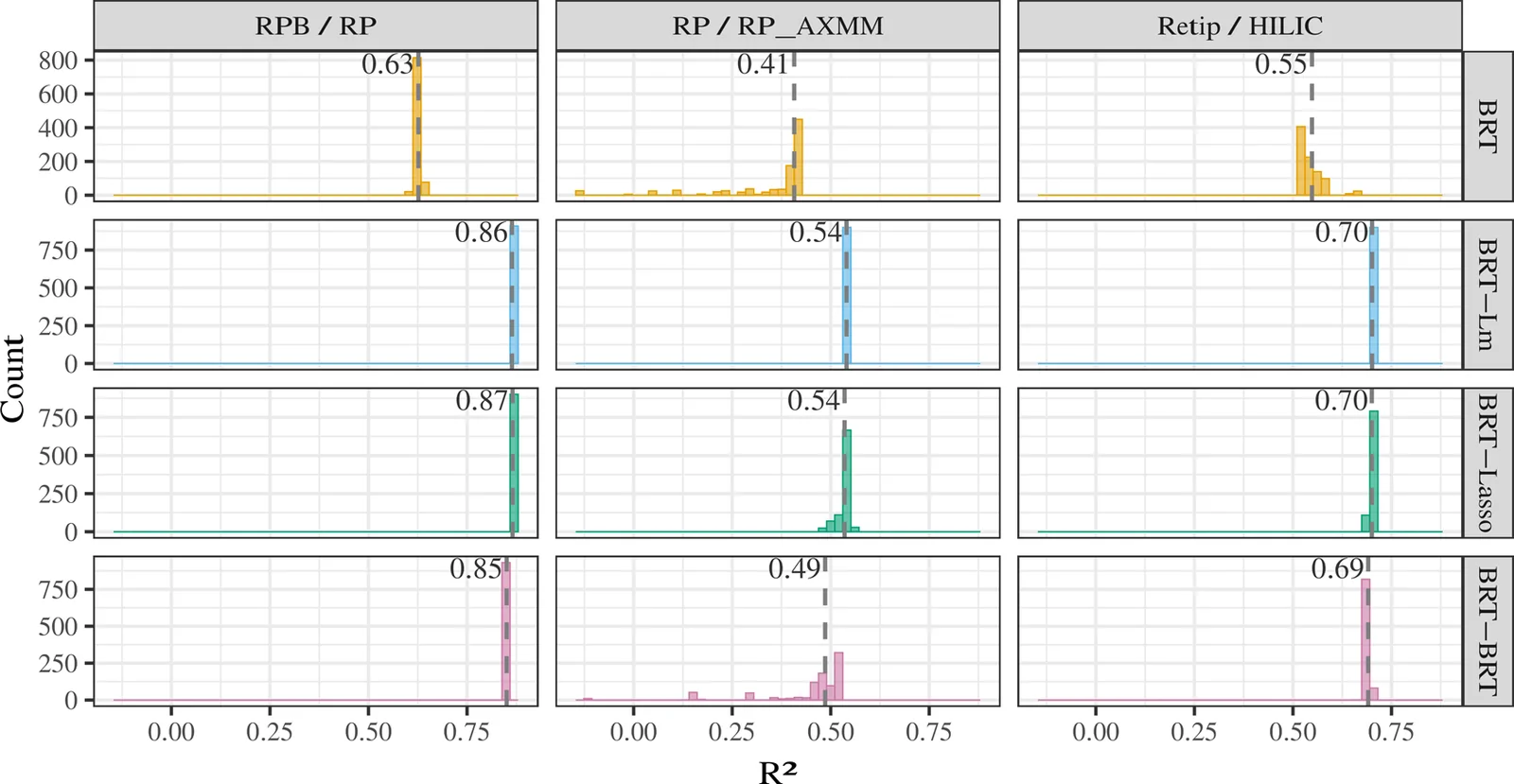
Adjustment-Performance *R* ^2^ distributions across adjustment model types with training set size *n* = 25 and base type BRT. Subsets have been drawn using the SMmax selective measuring strategy and the process was repeated 1000 times using different seeds. Only the middle 90% of the data is shown to improve readability. The medians use the full data, including omitted 5% tails.

Results for all selection strategies with a BRT base model are shown in Supporting Information Figure S7, results for Lasso base models in Figure S8, and the corresponding *R* ^2^ distributions for *n* = 25 in Figures S9–S12.

### Adjustment Validation

To further validate the adjustment approach, we selected 25 metabolites from an earlier version of the RP data set (DSNr 1) using the SM1 selective measuring strategy and remeasured them under six chromatographic conditions (DSNr 6–11). The conditions differed in flow rate, temperature, and gradient steepness and are described in Table 1. The RT correlations between the base RP data set and these six variants are shown in Supporting Information Figure S13. These measurements were used to adjust the model trained on the full RP data set (DSNr 1). Performance was evaluated on 22 validation metabolites (DSNr 13–18) that were not part of the original RP data set, measured under the same conditions as the adjustment sets (DSNr 6–11). Fitting of the adjustment model was repeated 10 times with different random seeds to obtain performance distributions. RTs were matched by INCHIKEY for the adjustment model training. Median observed *R* ^2^ values (across seeds) for all six adjusted models are shown in Table 3. We report performance for BRT models trained from scratch (BRT) on the 25 remeasured metabolites and BRT models trained on the full RP data set and adjusted with the 25 remeasured metabolites (BRT-Lasso). BRT-Lasso model adjustment outperformed BRT models trained from scratch on the 25 remeasured metabolites. Except for data set combination number 4, the adjustment yielded *R* ^2^ values ranging from 0.81–0.84, which is similar to the from-scratch training performance using the full RP data set (*R* ^2^ = 0.86).

**Table 3:** Median *R* ^2^ Values across Seeds for the Adjustment Validation Data Sets^a^

DCNr	Train	Test	BRT	BRT-Lasso
4	RP_Flat	RP_Val_Flat	0.54	0.74
5	RP_FR25_Flat	RP_Val_FR25_Flat	0.55	0.84
6	RP_Steep	RP_Val_Steep	0.56	0.82
7	RP_T25_Flat	RP_Val_T25_Flat	0.76	0.84
8	RP_T25_FR25_Flat	RP_Val_T25_FR25_Flat	0.63	0.83
9	RP_T25_FR25_Steep	RP_Val_T25_FR25_Steep	0.60	0.81

a DCNr: Data set combination number (see Table 2). Train: data set for training the adjustment model. Test: data set for testing the adjusted model. BRT: *R* ^2^ for BRT model trained from scratch on the 25 remeasured metabolites. BRT-Lasso: *R* ^2^ for BRT model trained on the full RP data set and adjusted with the 25 remeasured metabolites.

### Comparison with Other Methods

To place FastRet in the context of existing tools, we benchmarked it against four published methods on identical train/test splits (Tables 4 and 5): Retip^9^ with a gradient-boosted-tree (XGBoost) backend, Retip^9^ with a neural-network (Keras) backend, both on CDK descriptors; DeepGCN-RT,^14^ a graph neural network pretrained on the SMRT data set; and a PredRet^15^-like projection, a monotone RT spline fed by a FastRet base model (see Table 5). Trained from scratch on the full training sets, all descriptor- and graph-based methods are competitive: FastRet-BRT and the more heavily tuned Retip-xgboost differ only marginally. To check that this small gap reflects hyperparameter-search effort, we trained FastRet-BRT with its built-in grid search (GS) enabled as a control (FastRet-BRT-GS). It reproduces Retip-xgboost across all data sets (e.g., *R* ^2^ = 0.74 vs 0.74 on RP-AXMM), whereas FastRet-BRT with its fast default settings, which are used throughout this paper, trails both.

**Table 4:** Benchmark of FastRet against Retip^9^ and DeepGCN-RT,^14^ Trained from Scratch on Each Data Set’s Full Training Split^a^

	FastRet	FastRet	FastRet	Retip	Retip	DeepGCN
Data set	-Lasso	-BRT	-BRT-GS	-xgb	-keras	-RT
RP	0.74 ± 0.02	0.86 ± 0.00	**0.90** ± **0.00**	**0.91** ± **0.00**	0.73 ± 0.04	0.85 ± 0.02
RPB	0.76 ± 0.00	0.87 ± 0.00	0.90 ± 0.00	**0.91** ± **0.00**	0.71 ± 0.02	0.84 ± 0.02
RP-AXMM	0.47 ± 0.06	0.66 ± 0.00	**0.74** ± **0.00**	**0.74** ± **0.00**	0.29 ± 0.19	0.66 ± 0.02
HILIC	0.73 ± 0.01	**0.81** ± **0.00**	**0.81** ± **0.00**	0.80 ± 0.00	–0.65 ± 0.98	**0.81** ± **0.02**
Retip-HILIC	0.50 ± 0.06	0.54 ± 0.00	**0.63** ± **0.00**	**0.62** ± **0.00**	–11 ± 12	0.51 ± 0.02

a Cells are the mean test-set *R* ^2^ ± SD over five random seeds; the five rows are the five data sets. Bold marks every method within 0.01 *R* ^2^ of the row’s best. FastRet-BRT and Retip-xgboost both fit boosted trees using rcdk-computed CDK descriptors, so FastRet-BRT-GS, FastRet-BRT with its built-in grid search enabled (train_frm­(tune_grid = “small”)), reproduces Retip-xgboost. Runtimes are reported in Supporting Information Table S11.

**Table 5:** Benchmark of FastRet against PredRet^15^-like, DeepGCN-RT^14^ and Retip^9^ in the Adjustment Setting: A Base Model is Transferred to a New Setup from *N* = 25 or *N* = 50 SMmax-Selected Compounds^a^

		FastRet	FastRet	PredRet-like	FastRet	DeepGCN	Retip	Retip
*N*	Data set	-BRT-Lasso	-BRT-LM	(GAM)	-TFS25	-RT	-xgb	-keras
25	RPB → RP	**0.87** ± **0.00**	**0.86** ± **0.00**	**0.87** ± **0.00**	0.63 ± 0.00	0.67 ± 0.12	–0.02 ± 0.00	–20 ± 37
	RP → RPB	0.81 ± 0.05	**0.86** ± **0.00**	**0.86** ± **0.00**	0.64 ± 0.03	0.73 ± 0.04	–0.05 ± 0.01	–1.68 ± 1.34
	RP → RP-AXMM	**0.53** ± **0.02**	**0.54** ± **0.00**	0.49 ± 0.01	0.37 ± 0.13	0.33 ± 0.23	–0.14 ± 0.02	–4.13 ± 2.16
	Retip-HILIC → HILIC	**0.70** ± **0.00**	**0.70** ± **0.00**	**0.70** ± **0.01**	0.54 ± 0.01	–0.08 ± 0.32	0.01 ± 0.01	–5.67 ± 5.22
	HILIC → Retip-HILIC	**0.59** ± **0.00**	**0.58** ± **0.00**	0.56 ± 0.00	0.46 ± 0.03	–0.31 ± 0.75	–0.49 ± 0.06	–44 ± 30
50	RPB → RP	**0.87** ± **0.00**	**0.87** ± **0.00**	**0.87** ± **0.00**	0.77 ± 0.02	0.36 ± 0.35	0.71 ± 0.03	–0.30 ± 0.41
	RP → RPB	**0.85** ± **0.03**	**0.86** ± **0.00**	**0.85** ± **0.00**	0.68 ± 0.04	0.67 ± 0.13	0.71 ± 0.03	–0.93 ± 1.03
	RP → RP-AXMM	**0.65** ± **0.01**	0.52 ± 0.00	0.50 ± 0.00	0.57 ± 0.05	0.06 ± 0.27	**0.66** ± **0.01**	–0.75 ± 0.79
	Retip-HILIC → HILIC	**0.74** ± **0.01**	0.70 ± 0.00	0.72 ± 0.00	0.70 ± 0.02	0.71 ± 0.07	0.72 ± 0.01	–2.30 ± 3.10
	HILIC → Retip-HILIC	**0.58** ± **0.01**	**0.57** ± **0.01**	0.55 ± 0.01	0.47 ± 0.04	0.51 ± 0.07	0.56 ± 0.02	–45 ± 38

a Cells are the mean test-set *R* ^2^ ± SD over ten random seeds; each *N* block lists the five source → target data set pairs. Bold marks every method within 0.01 *R* ^2^ of the row’s best. FastRet-BRT-Lasso (descriptor-aware) and -BRT-LM (linear, base-RT only) adjust a base BRT model, the PredRet-like column projects retention times through a monotone spline, FastRet-TFS25 trains from scratch on the few compounds, DeepGCN-RT fine-tunes the SMRT graph network, Retip-xgboost grid-searches on them and Retip-keras trains a neural network on them. The PredRet-like column is not the published PredRet: we keep its monotone-spline projection of retention times between data sets, but because that method requires every query compound to have been measured in the base data set (ours were not) we feed the spline base-data set retention times predicted by a FastRet BRT model from chemical descriptors instead of measured ones. The from-scratch setting is in Table 4; runtimes are in Supporting Information Table S11.

The results for the low-data regime, i.e., adjustment for a new setup from only 25 or 50 selected compounds, are shown in Table 5. FastRet’s adjustment is the strongest structure-based method and, together with the PredRet-like projection, the only one applicable to the cross-library HILIC pair, where the target compounds were never measured in the base setup. Methods trained from scratch on so few compounds perform poorly: Retip’s grid search degenerates to near-constant predictions, and DeepGCN-RT, whose authors fine-tune on transfer sets of roughly 70–410 compounds rather than 25, remains competitive on the reversed-phase pairs but turns negative on the HILIC transfer. Among the three adjustment variants, a linear fit on the base RT alone (FastRet-BRT-LM) is already competitive, the monotone-spline projection (PredRet-like) helps only on the highly correlated reversed-phase pairs and can degrade where elution order is less conserved, and the descriptor-aware Lasso adjustment is best once more anchor compounds are available (Table 5, *N* = 50 rows). FastRet is also the most economical in compute, training and predicting in one to two seconds on a single core, whereas the competing tools require 16 cores and tens (Retip) to thousands (DeepGCN-RT) of seconds. Every method was timed on the identical workstation of Supporting Information Table S8, with the full breakdown given in Table S11.

Beyond these head-to-head comparisons on our in-house data, we further evaluated the generalization performance of FastRet using 14 publicly available data sets from the RepoRT repository^20^ (12 RP and two Amide-HILIC data sets from independent laboratories). Models trained from scratch using the shared compounds achieved a median held-out *R* ^2^ of 0.60 (RP) and 0.58 (HILIC). Similarly, adapting an in-house model using only 25–50 remeasured compounds yielded comparable performance (Supporting Information Table S12).

These results also define the applicability domain of FastRet and highlight its principal limitation. For a chromatographic system that differs substantially from those studied here in separation mechanism, stationary phase or gradient, the base model alone cannot be expected to be predictive, and compounds measured on that system are required, either a full training set or, at minimum, a selectively measured adjustment set. Per-column results vary accordingly, from values comparable with our in-house data down to negative held-out *R* ^2^ for individual external columns (Supporting Information Table S12).

## Conclusion

FastRet combines three practically relevant components for RT modeling: training from scratch, adjusting an existing model to account for modified chromatographic conditions, and selective measuring to prioritize which metabolites to remeasure to limit training data. These three tasks and RT prediction can be performed through a user-friendly and accessible web interface as illustrated in the Supporting Information Figures S14–S17. An example of using FastRet for the annotation of two features is given in the Supporting Information Figure S18.

Across data sets, BRT generally achieved higher performance than Lasso when larger training sets were available, while both methods performed similarly at small training sizes. Performance differed by separation mechanism, with RP showing the strongest prediction accuracy and RP-AXMM being more challenging, which is consistent with the mixed-mode retention behavior of RP-AXMM.

Selective measuring consistently improved performance compared to random metabolite selection when training with small subsets. This supports the idea that targeted remeasurement of informative metabolites is more efficient than random selection under limited measurement budgets. In particular, SM1 showed the best overall gains versus random selection in all three evaluated data set combinations.

Because FastRet, like any descriptor-based QSRR model, can only be expected to predict reliably for compound classes represented in its training data, the composition of the training set matters as much as its size. This is reflected in the *R* ^2^ distributions of Figure 7 (which displays the central 90% of each distribution): across the 1000 training sets of 25 randomly chosen RP metabolites the test-set *R* ^2^ spans −0.53 to 0.81 (interquartile range 0.43–0.65) and falls below zero for the most narrowly composed subsets, whereas selective measuring confines it to 0.54 to 0.72 (interquartile range 0.61–0.62).

An important practical result is that model adjustment with a small number of remeasured metabolites (25 in our experiments) provided larger performance gains than training from scratch on equally small, randomly selected subsets. In other words, selective measuring improves from-scratch training, but leveraging an existing base model and adjusting it is typically even more effective under the same remeasurement budget. This was evident both in the paired-data set experiments and in the independent validation using remeasured RP variants, where adjustment with Lasso models outperformed adjustment with BRT models trained only on the 25 remeasured metabolites. Accordingly, for adjustment with small remeasurement sets such as the 25-metabolite setting studied here, we recommend using Lasso for the adjustment step rather than BRT.

To probe generalization beyond our own laboratory, we additionally validated FastRet on 12 RP and two Amide-HILIC data sets from the RepoRT repository,^20^ both trained from scratch on the shared compounds and adjusted from only 25 to 50 remeasured compounds (reported in Comparison with Other Methods section and Supporting Information Table S12); a laboratory operating a different instrument can thus obtain a working model from a handful of measurements instead of a full retraining. Performance nonetheless varied across setups: predictions degraded for compound classes underrepresented in our training data, and a reversed-phase column operated with a much longer gradient than any in our training data remained hard to predict. Consistent with the composition argument above, FastRet therefore generalizes across laboratories and platforms as a *method* to be retrained or adjusted per chromatographic system—typically from only a handful of remeasured compounds—rather than as a single portable model. The independent validation on 22 unseen metabolites per condition further supports robust adjustment within a laboratory. Accordingly, generalization claims should be interpreted within the scope of this study: our in-house RP, RP-AXMM, and HILIC data, one external HILIC data set compared in depth, and a further 12 RP and two HILIC RepoRT data sets benchmarked against FastRet’s BRT and Lasso adjustment models.

Finally, model quality remains constrained by descriptor quality and measurement coverage, and by how far the target chromatographic system departs from the one the base model was built on. Improvements in molecular descriptors and expanded high-quality RT libraries are therefore likely to directly improve prediction and adjustment performance in future FastRet releases. As a brief outlook, promising next steps are adjustment with multiple base data sets and using predicted (instead of matched) base-model RTs for adjustment-model training when paired measurements are unavailable.

## Supplementary Material



## Data Availability

The FastRet R package is available on CRAN at https://cran.r-project.org/package=FastRet and as source code on GitHub at https://github.com/spang-lab/FastRet, released under the GPL-3 license. All in-house measurement data underlying this study are included in that repository (Measurements_v10P.xlsx), and the presented figures and tables can be reproduced using the code in the repository; the cross-laboratory validation additionally uses the public RepoRT data sets.^20^ The functionality of FastRet is also available through a web interface hosted at https://fastret.spang-lab.de and bundled in the R package (launchable locally via FastRet::start_gui­()). A step-by-step tutorial covering its four modes, with two annotated slides per mode, is provided in Figures S14–S17 of the Supporting Information; an online usage tutorial is additionally available at https://spang-lab.github.io/FastRet/. An application example using FastRet’s predicted retention times to narrow down the candidate hits of an HMDB accurate-mass search for two peaks measured in a NIST plasma extract is shown in Figure S18.

## Notes

Metabolite-presence heatmap across data sets (Figure S1); descriptor-signature heatmaps of the FastRet models (Lasso coefficients and XGBoost gain) (Figure S2); selective-measuring performance, BRT models (MAE) (Figure S3); selective-measuring performance, Lasso models (*R* ^2^) (Figure S4); selective-measuring performance, Lasso models (MAE) (Figure S5); RT correlation between the three core data set combinations (Figure S6); adjustment performance (*R* ^2^) for BRT base models (Figure S7); adjustment performance (*R* ^2^) for Lasso base models (Figure S8); adjustment *R* ^2^ distribution (*n* = 25, BRT, Random selection) (Figure S9); adjustment *R* ^2^ distribution (*n* = 25, BRT, SMinf selection) (Figure S10); adjustment *R* ^2^ distribution (*n* = 25, BRT, SM0 selection) (Figure S11); adjustment *R* ^2^ distribution (*n* = 25, BRT, SM1 selection) (Figure S12); RT correlation for model adjustment across RP variants (Figure S13); web-GUI Train tab (Figure S14); web-GUI Select (selective measuring) tab (Figure S15); web-GUI Adjust tab (Figure S16); web-GUI Predict tab (Figure S17); application of FastRet (Figure S18); liquid-chromatography columns (Table S1); mobile-phase eluents (Table S2); chromatographic gradients (Table S3); 25 SM1-selected medoid metabolites (Table S4); 22-compound independent validation set (Table S5); families of the CDK descriptors used by FastRet (Table S6); pairwise method comparisons (mean paired *R* ^2^ difference, 95% CI, paired *t*-test) (Table S7); computational environment (OS, R, Java, package versions) (Table S8); FastRet training runtimes with/without CV performance estimation (Table S9); per-combination *R* ^2^ performance summary (mean ± SD, 1000 seeds) (Table S10); training/prediction runtimes versus Retip, DeepGCN-RT and PredRet (Table S11); cross-lab external validation on RepoRT data sets (Table S12); chemical-descriptor computation with caching (Listing S1); training a model from scratch (Listing S2); selecting *k* compounds to remeasure (Listing S3); adjusting a base model to new conditions (Listing S4); clipping predicted retention times (Listing S5); predicting retention times from a model (Listing S6); estimating benchmark performance (Listing S7); constructing paired data set combinations (Listing S8); Lasso regression model family (Note S1); and Boosted Regression Trees model family (Note S2) (PDF)
