# Supplementary material for: FastRet: Fast and Simple Retention Time Prediction in Liquid Chromatography
Source: J Chem Inf Model. 2026 Aug 12;66(16):10412–25. doi: 10.1021/acs.jcim.6c01344 (PMC13508770; doi:10.1021/acs.jcim.6c01344)
Supplement: Supplementary file 1 [file ci6c01344_si_001.pdf]

Supporting Information

FastRet: Fast and Simple Retention Time Prediction in Liquid Chromatography

Fadi Fadil†[1,2], Tobias Schmidt†[3], Christian Amesoeder[3], Simon Heckscher[1], Marian Schoen[3], Wolfram Gronwald[1], Peter J. Oefner[1], Rainer Spang[3], and Katja Dettmer*[1]

[1] Institute of Functional Genomics, University of Regensburg, 93053 Regensburg, Germany
[2] Institute of Medical Microbiology, Immunology and Hygiene, Uniklinik Köln, 50935 Köln, Germany
[3] Department of Statistical Informatics, University of Regensburg, 93053 Regensburg, Germany

†These authors contributed equally to this work.
*Corresponding author: katja.dettmer@ur.de

Contents

- Supplementary figures (Figures S1–S18): data set heatmap, descriptor signatures, selective-measuring and adjustment performance curves, adjustment R^2-distribution histograms, RT-correlation pair plots, web-GUI screenshots, and examples for the application of FastRet.
- Supplementary tables (Tables S1–S12): chromatographic columns, eluents and gradients; selected medoids and validation set; method-comparison, external-method-benchmark and cross-lab validation results; per-combination performance summary; runtimes; descriptor families; and computational environment.
- Pseudocode listings (Listings S1–S8) for the core FastRet procedures.
- Supplementary notes (Notes S1–S2): additional details on the Lasso and Boosted Regression Trees model families.

Contents

Figures

Figure S1: Metabolite-presence heatmap across data sets S5

Figure S2: Descriptor-signature heatmaps of the FastRet models (Lasso coefficients and XGBoost gain) S6

Figure S3: Selective-measuring performance, BRT models (MAE) S6

Figure S4: Selective-measuring performance, Lasso models (R^2) S7

Figure S5: Selective-measuring performance, Lasso models (MAE) S7

Figure S6: RT correlation between the three core data set combinations S7

Figure S7: Adjustment performance (R^2) for BRT base models S9

Figure S8: Adjustment performance (R^2) for Lasso base models S10

Figure S9: Adjustment R^2 distribution ($n = 25$, BRT, Random selection) S10

Figure S10: Adjustment R^2 distribution ($n = 25$, BRT, SMinf selection) S11

Figure S11: Adjustment R^2 distribution ($n = 25$, BRT, SM0 selection) S11

Figure S12: Adjustment R^2 distribution ($n = 25$, BRT, SM1 selection) S12

Figure S13: RT correlation for model adjustment across RP variants S13

Figure S14: Web-GUI Train tab S14

Figure S15: Web-GUI Select (selective measuring) tab S15

Figure S16: Web-GUI Adjust tab S16

Figure S17: Web-GUI Predict tab S17

Figure S18: Application of FastRet S17

Tables

Table S1: Liquid-chromatography columns S18

Table S2: Mobile-phase eluents S18

Table S3: Chromatographic gradients S19

Table S4: 25 SM1-selected medoid metabolites S20

Table S5: 22-compound independent validation set S20

Table S6: Families of the CDK descriptors used by FastRet S21

Table S7: Pairwise method comparisons (mean paired R^2 difference, 95% CI, paired t-test) S22

Table S8: Computational environment (OS, R, Java, package versions) S23

Table S9: FastRet training runtimes with/without CV performance estimation S24

Table S10: Per-combination R^2 performance summary (mean $\pm$ SD, 1000 seeds) S24

Table S11: Training/prediction runtimes versus Retip, DeepGCN-RT and PredRet S25

Table S12: Cross-lab external validation on RepoRT data sets S26

Listings (pseudocode)

Listing S1: Chemical-descriptor computation with caching S26

Listing S2: Training a model from scratch S27
Listing S3: Selecting k compounds to remeasure S27
Listing S4: Adjusting a base model to new conditions S27
Listing S5: Clipping predicted retention times S28
Listing S6: Predicting retention times from a model S28
Listing S7: Estimating benchmark performance S28
Listing S8: Constructing paired data set combinations S29

Supplementary Notes

Note S1: Lasso regression model family S30
Note S2: Boosted Regression Trees model family S30

Figures

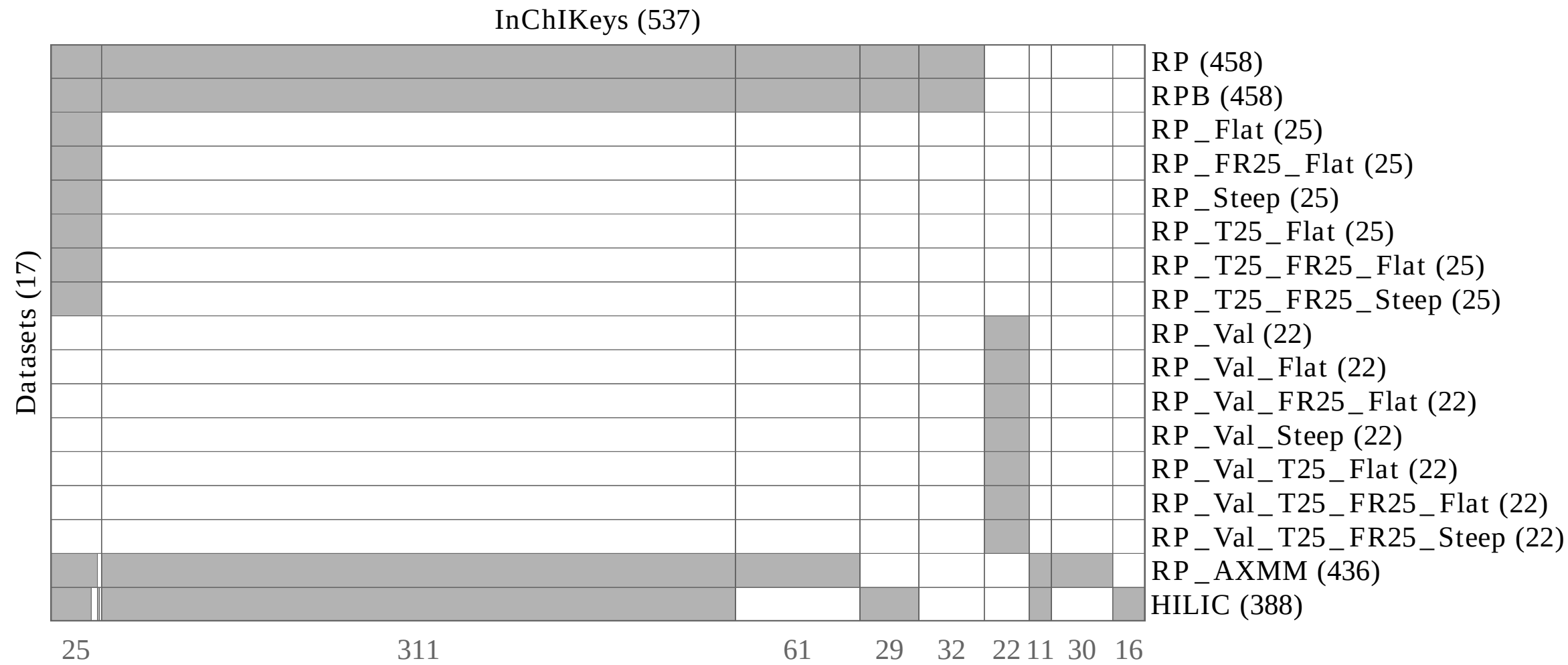

Figure S1: Heatmap visualizing the presence of compounds across data sets. Each row represents a data set, each column a set of compounds. A filled cell indicates that the compound set is present in the respective data set. Vertical lines are drawn to highlight groups of compounds shared across multiple data sets. The number of compounds in a group is given at the bottom. Data set IDs are given on the right. Data set HILIC-Retip is not shown, to improve readability.

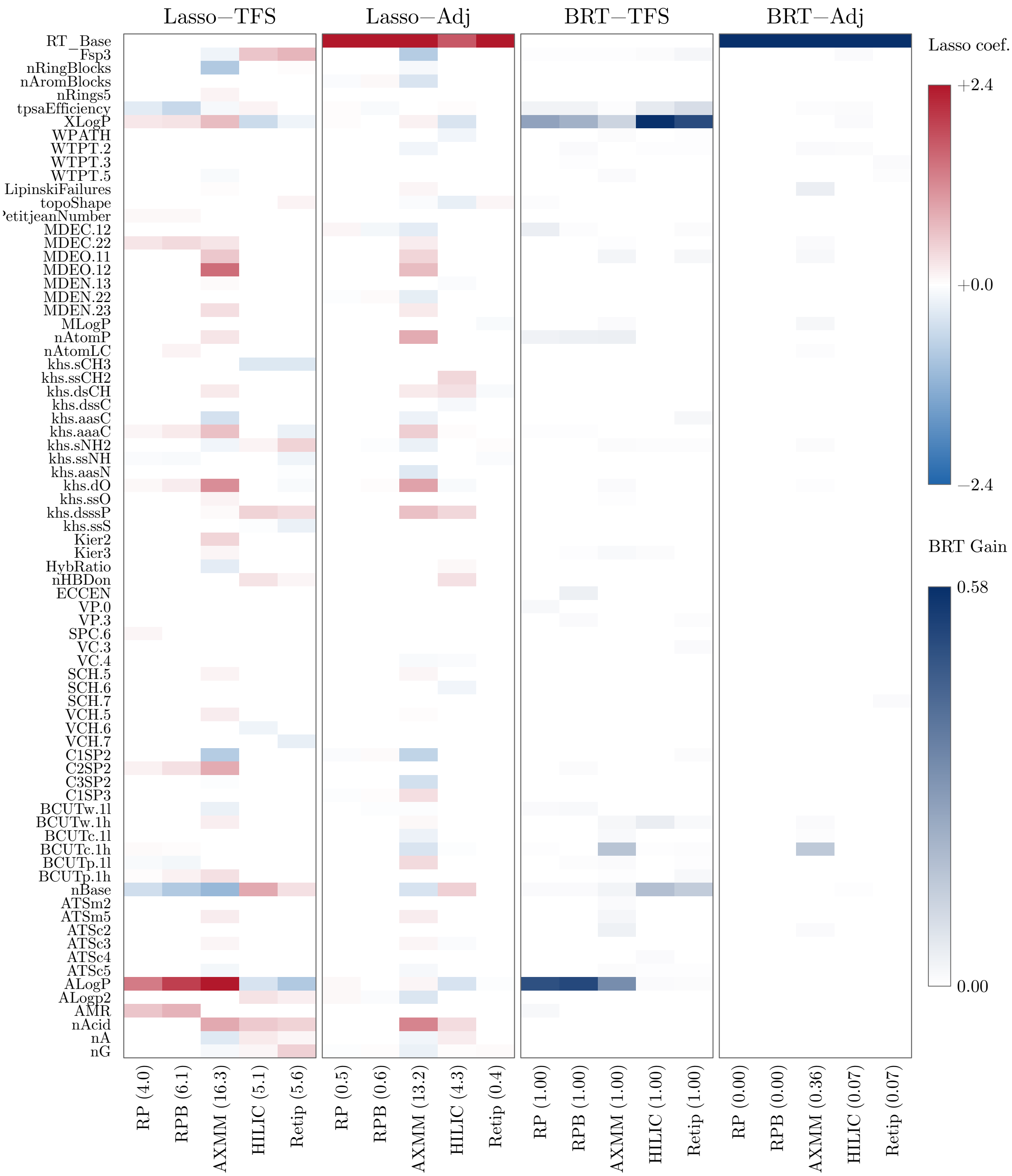

Figure S2: Descriptor signatures of FastRet models. Panels show standardized Lasso coefficients or XGBoost Gain for models trained from scratch (TFS) or as adjustment models (Adj). Columns are the five data sets. For adjustment models, the given data set is the target, with bases RPB→RP, RP→RPB, RP→RP-AXMM, Retip→HILIC, HILIC→Retip. The value after each data set is that model's sum of absolute standardized coefficients (Lasso) or total Gain (BRT). Rows show RT-base (used by Adjustment Models) and the 75 descriptors reaching |coef| ≥ 0.05 or Gain ≥ 0.01 in at least one model. Cells show the coefficient (red positive, blue negative) or Gain (darker=larger).

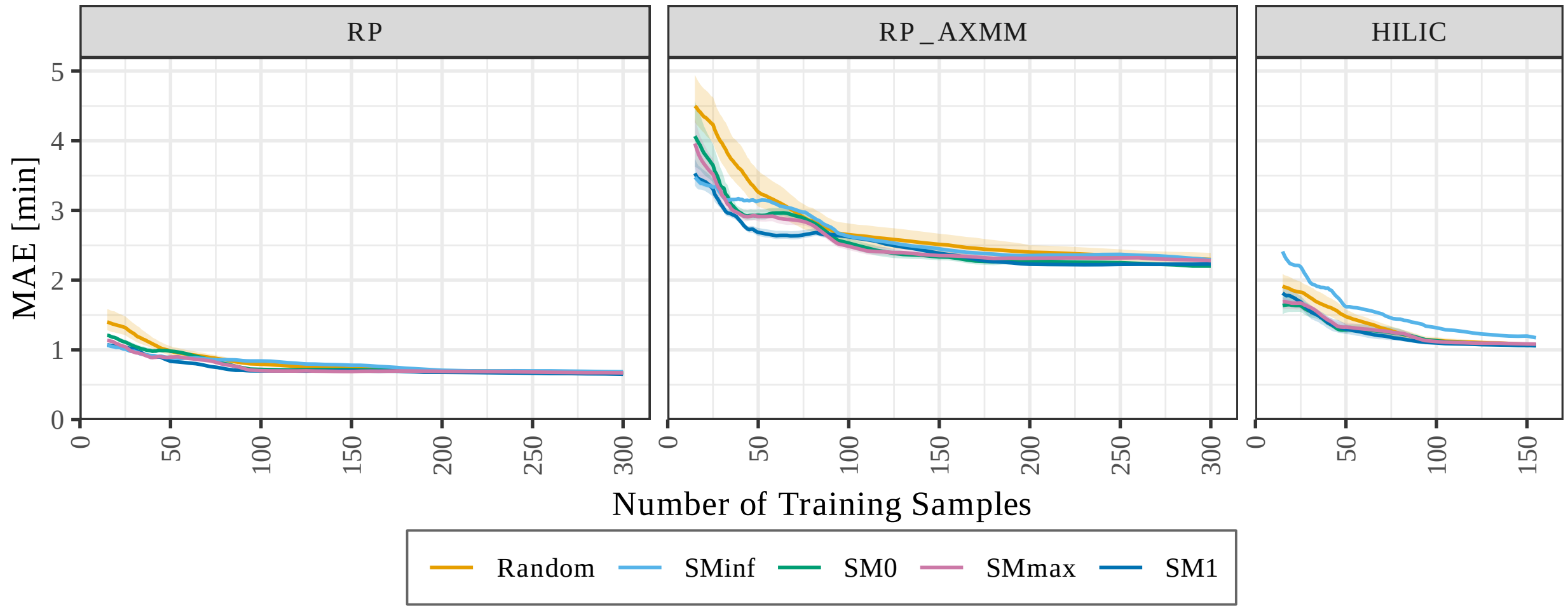

Figure S3: Selective-Measuring-Performance for BRT models (MAE) across training subset sizes for RP, RP-AXMM and HILIC data sets. For each parameter combination (selection strategy and training subset size), 10 repetitions were performed using different random seeds. The x-axis shows the number of compounds per training subset, and the y-axis shows the Mean Absolute Error (MAE) in RT prediction. Median and interquartile range calculations use the true MAE values. Different lines represent different selection strategies: Random (random subset selection), SMinf, SM0, SMmax, and SM1 (selective measuring variants). Solid lines show the median trend across the 10 repetitions per parameter combination, and shaded bands indicate the interquartile range across the 10 repetitions. The median and quartiles are smoothed using a moving average with a window size of 21 to improve readability.

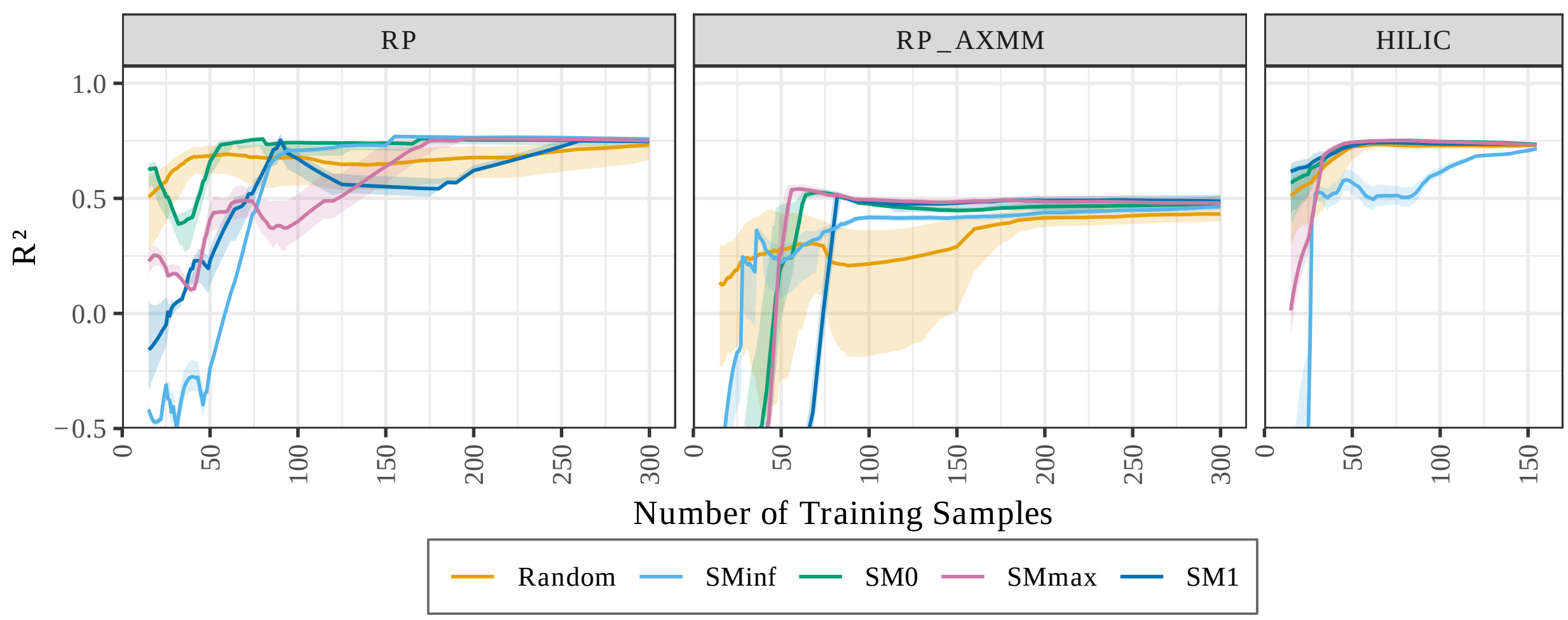

Figure S4: Selective-Measuring-Performance for Lasso models (R^2) across training subset sizes for RP, RP-AXMM and HILIC data sets. For each parameter combination (selection strategy and training subset size), 10 repetitions were performed using different random seeds. The x-axis shows the number of compounds per training subset, and the y-axis shows the R^2 performance. Different lines represent different selection strategies: Random (random subset selection), SMinf, SM0, SMmax, and SM1 (selective measuring variants). Solid lines show the median trend across the 10 repetitions per parameter combination, and shaded bands indicate the interquartile range across the 10 repetitions. The median and quartiles are smoothed using a moving average with a window size of 21 to improve readability.

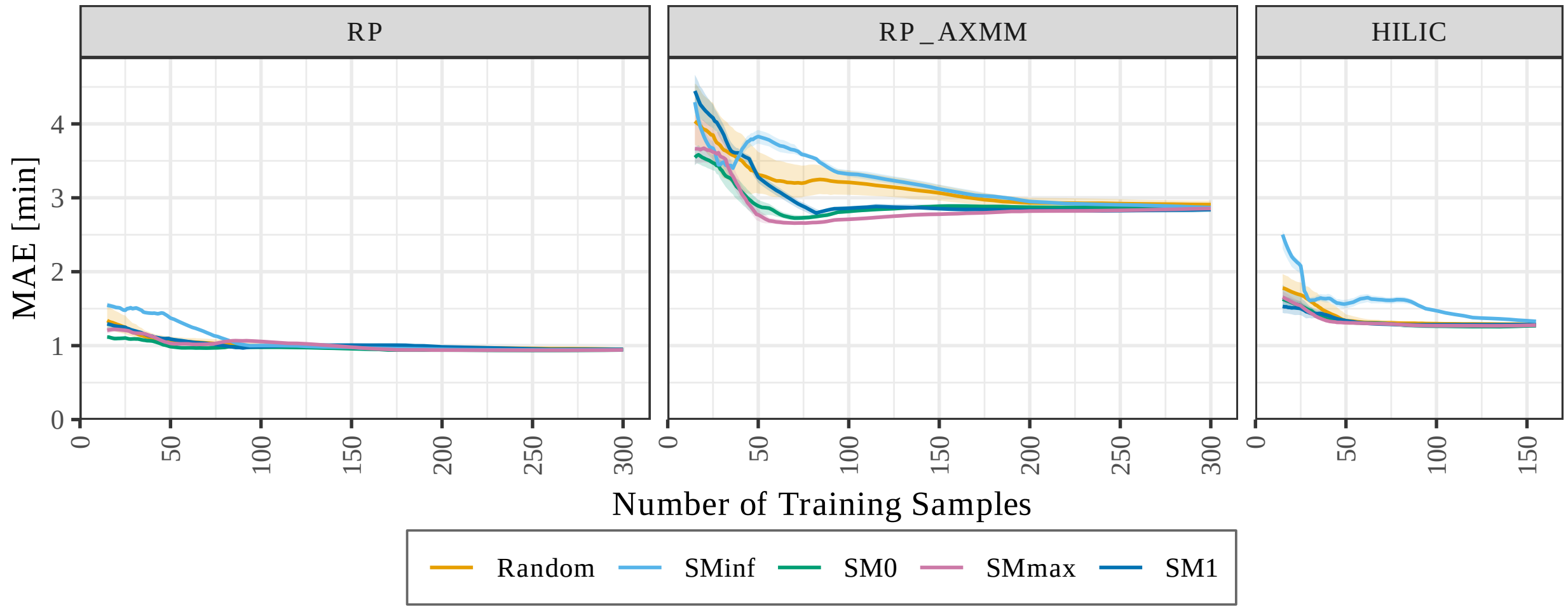

Figure S5: Selective-Measuring-Performance for Lasso models (MAE) across training subset sizes for RP, RP-AXMM and HILIC data sets. For each parameter combination (selection strategy and training subset size), 10 repetitions were performed using different random seeds. The x-axis shows the number of compounds per training subset, and the y-axis shows the Mean Absolute Error (MAE) in RT prediction. Median and interquartile range calculations use the true MAE values. Different lines represent different selection strategies: Random (random subset selection), SMinf, SM0, SMmax, and SM1 (selective measuring variants). Solid lines show the median trend across the 10 repetitions per parameter combination, and shaded bands indicate the interquartile range across the 10 repetitions. The median and quartiles are smoothed using a moving average with a window size of 21 to improve readability.

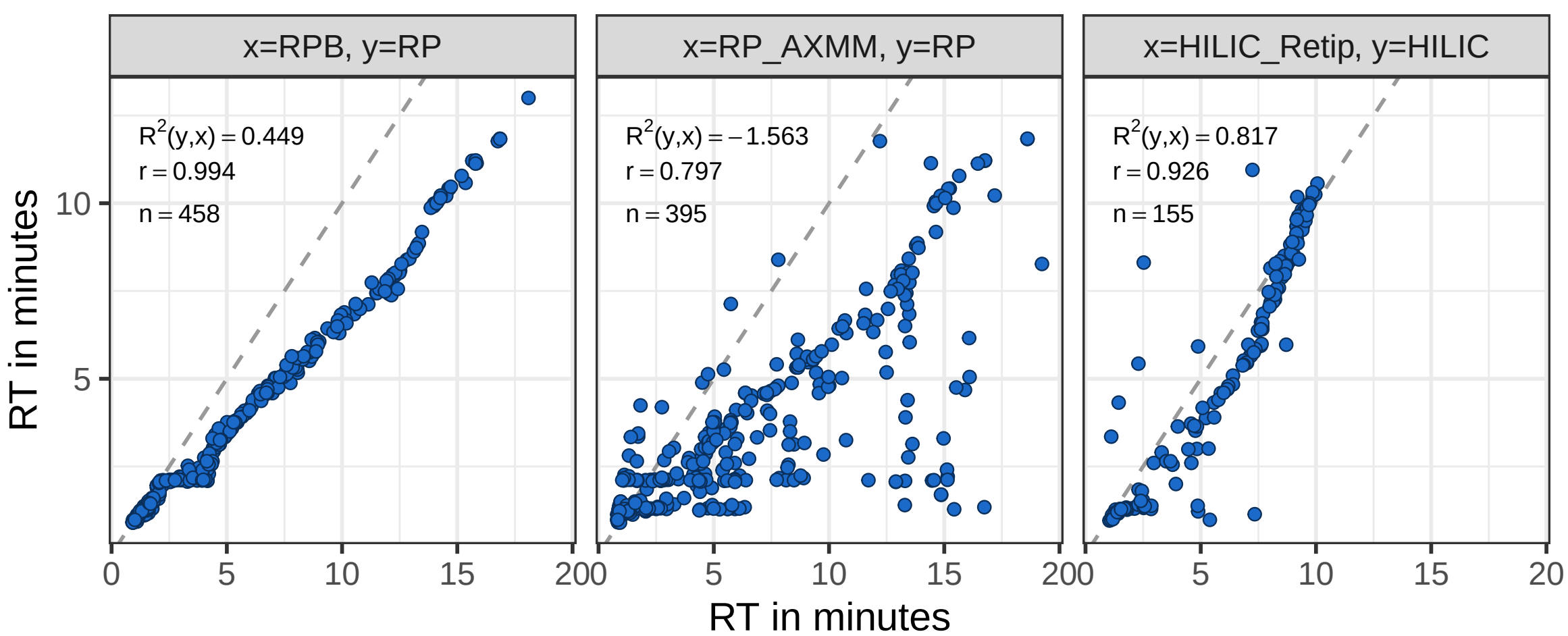

Figure S6: RT correlation between core data set combinations. Each panel shows a scatterplot of RTs for a paired data set combination, with all overlapping compounds plotted. Panel 1: x = RPB-Train, y = RP-Train. Panel 2: x = RP-AXMM-Train, y = RP-Train. Panel 3: x = HILIC-Retip-Train, y = HILIC-Train. The "-Train" suffix is not shown in the titles to improve readability.

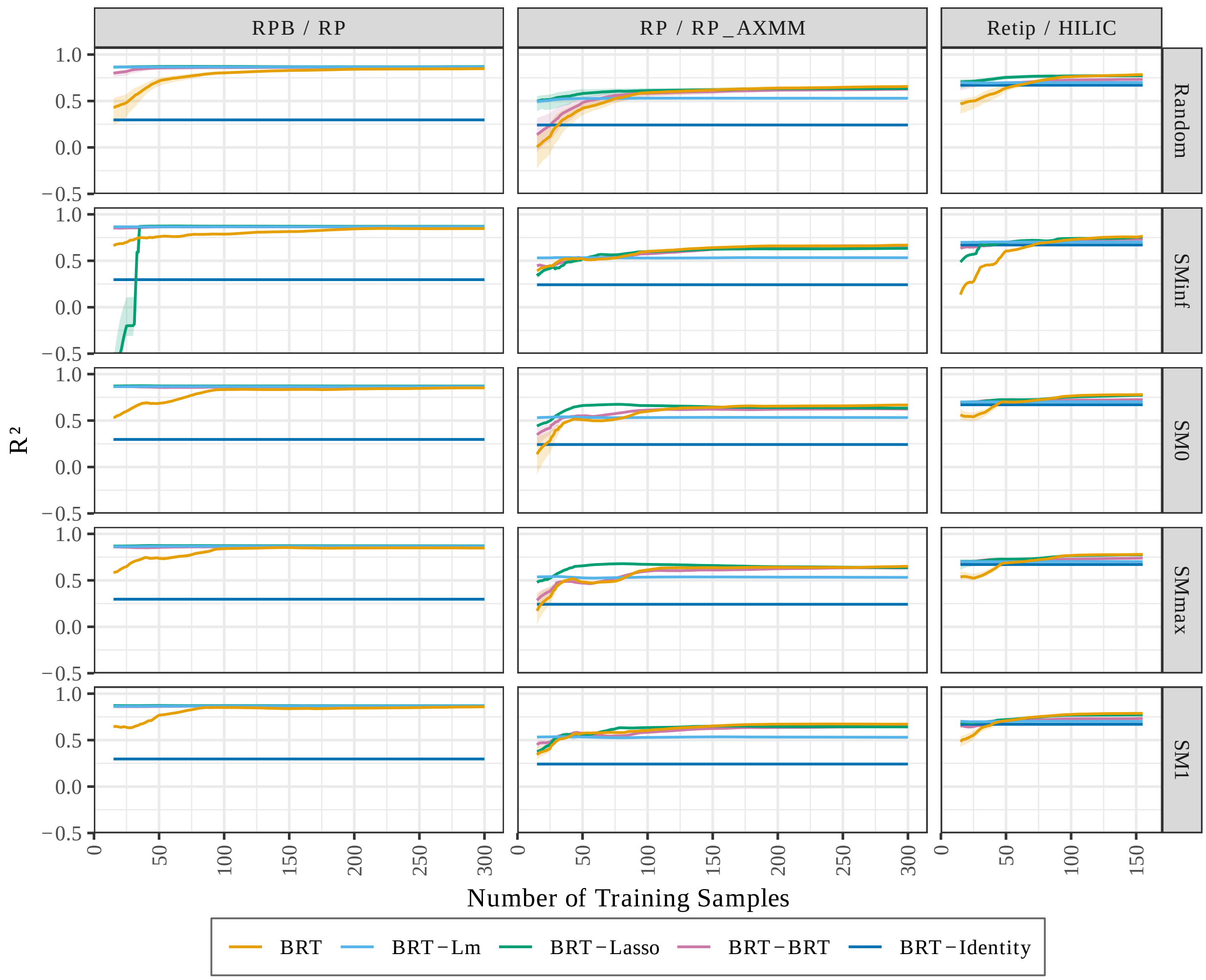

Figure S7: Adjustment performance (R^2) for BRT base models across training set sizes, with rows showing different selective measuring strategies (Random, SMinf, SM0, SMmax, SM1) and columns showing the three data set combinations (RPB/RP, RP/RP-AXMM, Retip/HILIC). For each adjustment model and subset size, 10 repetitions were performed using different random seeds. The base model was always trained on the full base data set. Lines represent adjustment model types: BRT (trained from scratch), BRT–Lm (linear adjustment), BRT–Lasso (Lasso adjustment), BRT–BRT (BRT adjustment), and BRT–Identity (base model without adjustment). Solid lines show the median across 10 repetitions; shaded bands indicate the interquartile range.

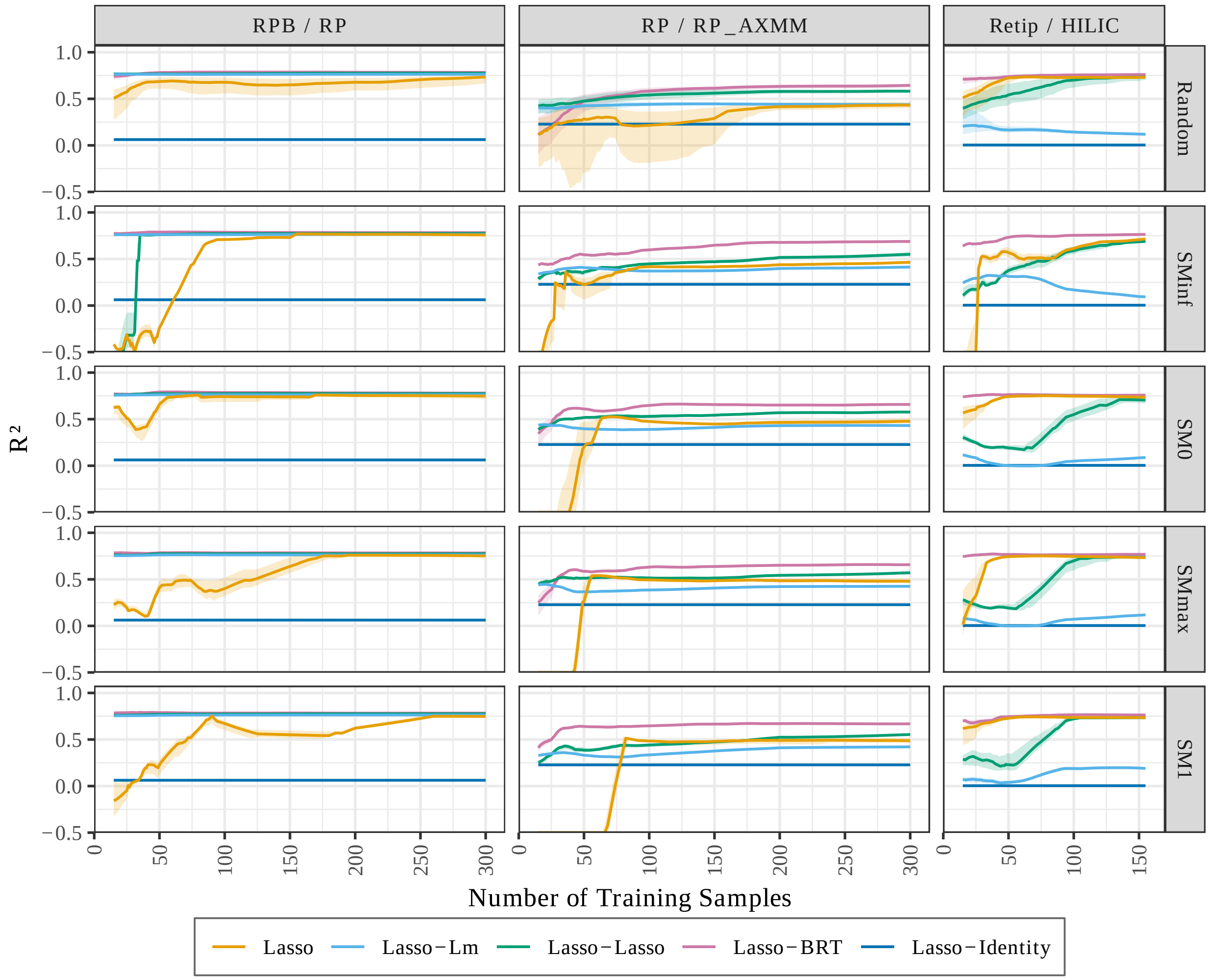

Figure S8: Adjustment performance (R^2) for Lasso base models across training set sizes, with rows showing different selective measuring strategies (Random, SMinf, SM0, SMmax, SM1) and columns showing the three data set combinations (RPB/RP, RP/RP-AXMM, Retip/HILIC). For each adjustment model and subset size, 10 repetitions were performed using different random seeds. The base model was always trained on the full base data set. Lines represent adjustment model types: Lasso (trained from scratch), Lasso–Lm (linear adjustment), Lasso–Lasso (Lasso adjustment), Lasso–BRT (BRT adjustment), and Lasso–Identity (base model without adjustment). Solid lines show the median across 10 repetitions; shaded bands indicate the interquartile range.

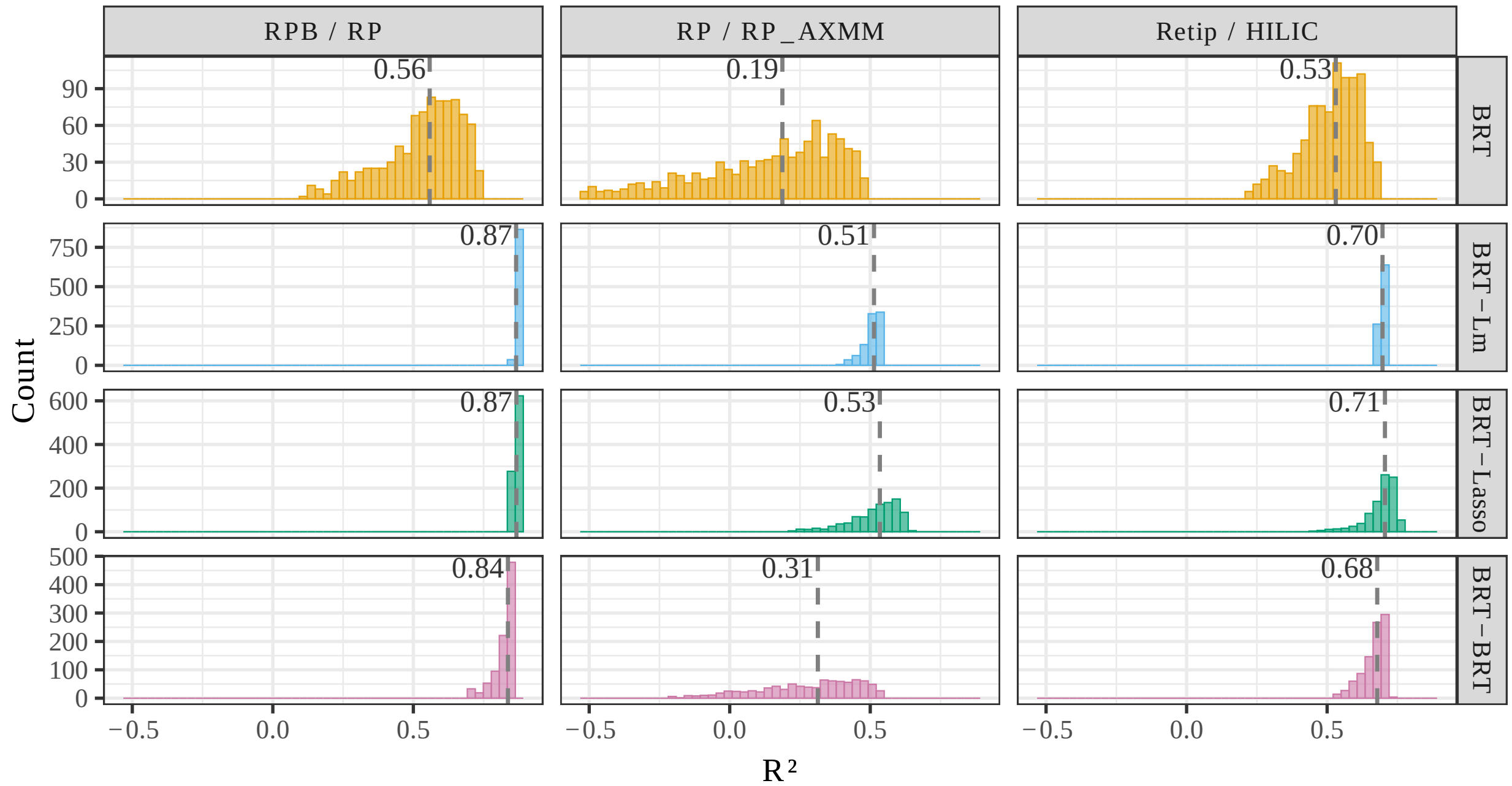

Figure S9: Adjustment-Performance R^2 distributions with $n = 25$ and base type BRT using Random selection. See Figure 9 for details on the plot layout.

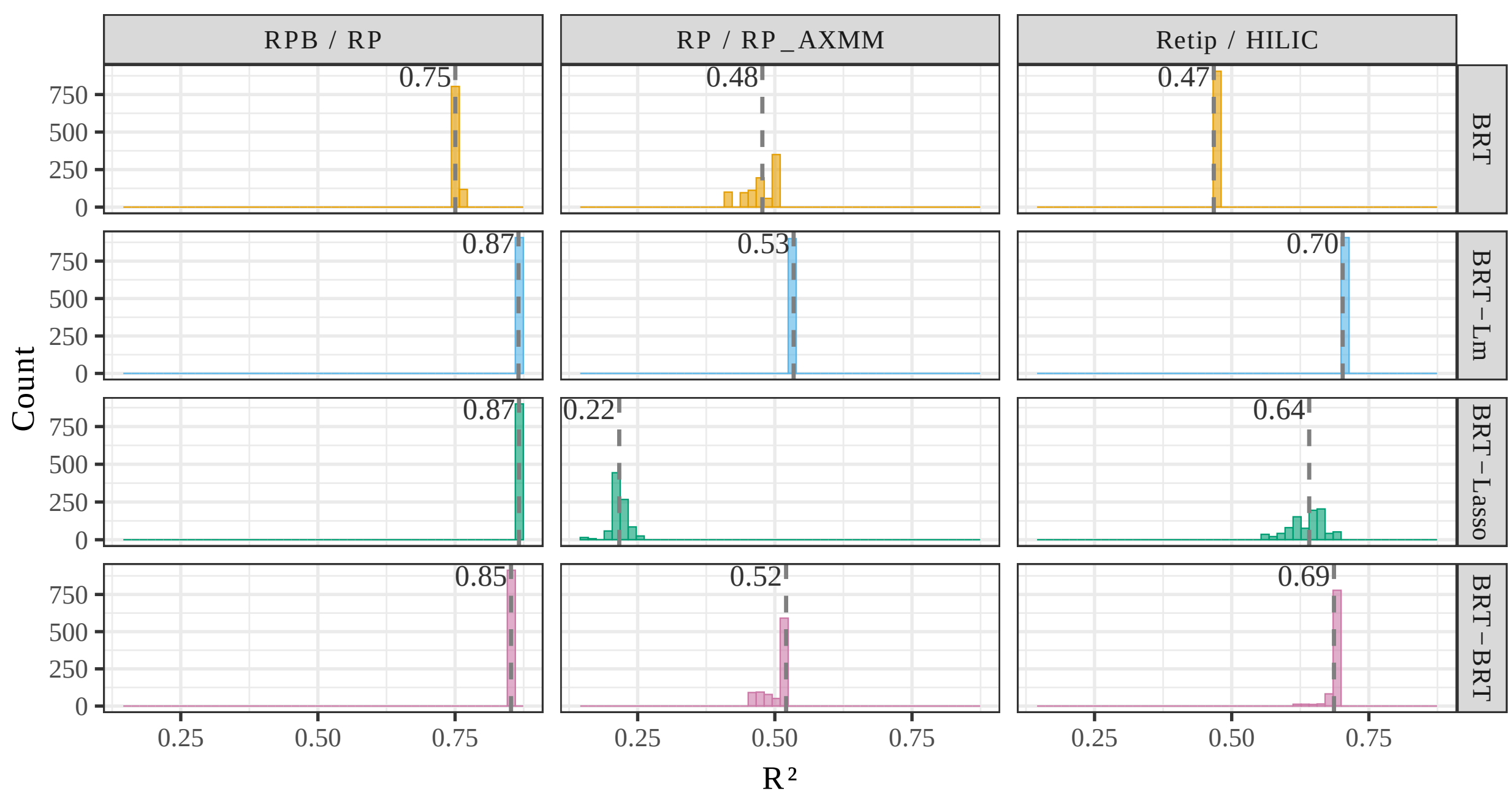

Figure S10: Adjustment-Performance R^2 distributions with $n = 25$ and base type BRT using SMinf selection. See Figure 9 for details on the plot layout.

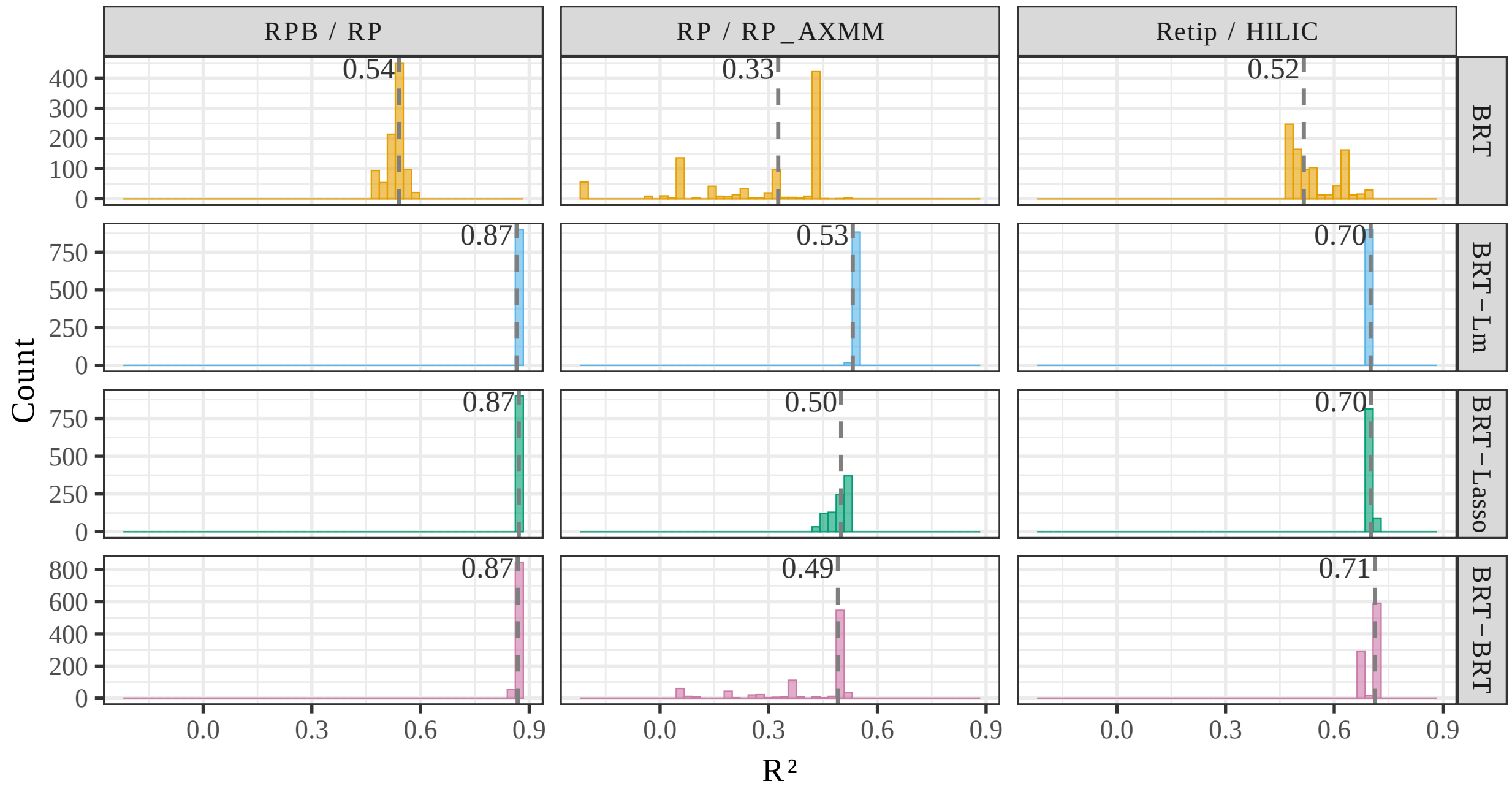

Figure S11: Adjustment-Performance R^2 distributions with $n = 25$ and base type BRT using SM0 selection. See Figure 9 for details on the plot layout.

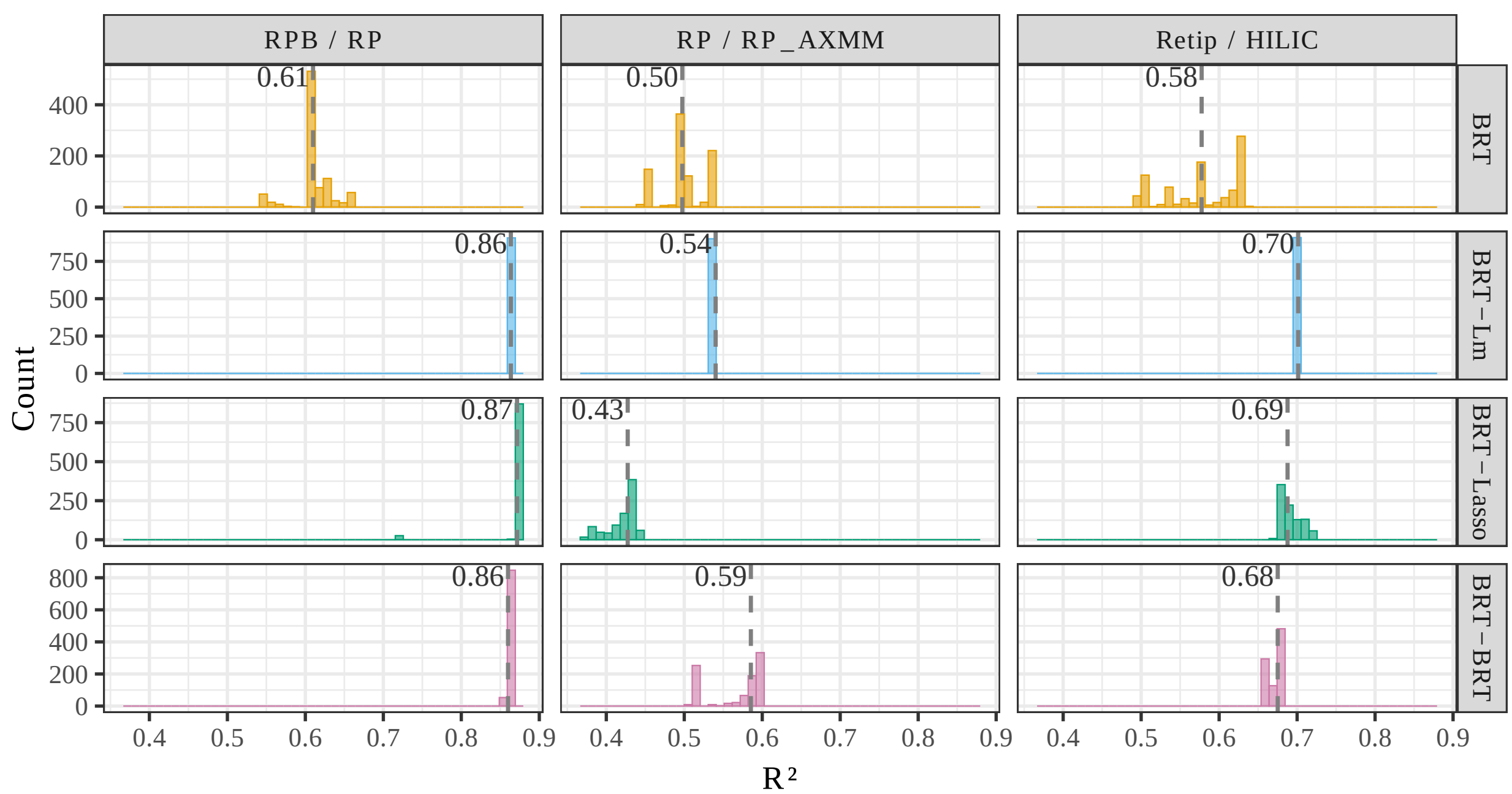

Figure S12: Adjustment-Performance R^2 distributions with $n = 25$ and base type BRT using SM1 selection. See Figure 9 for details on the plot layout.

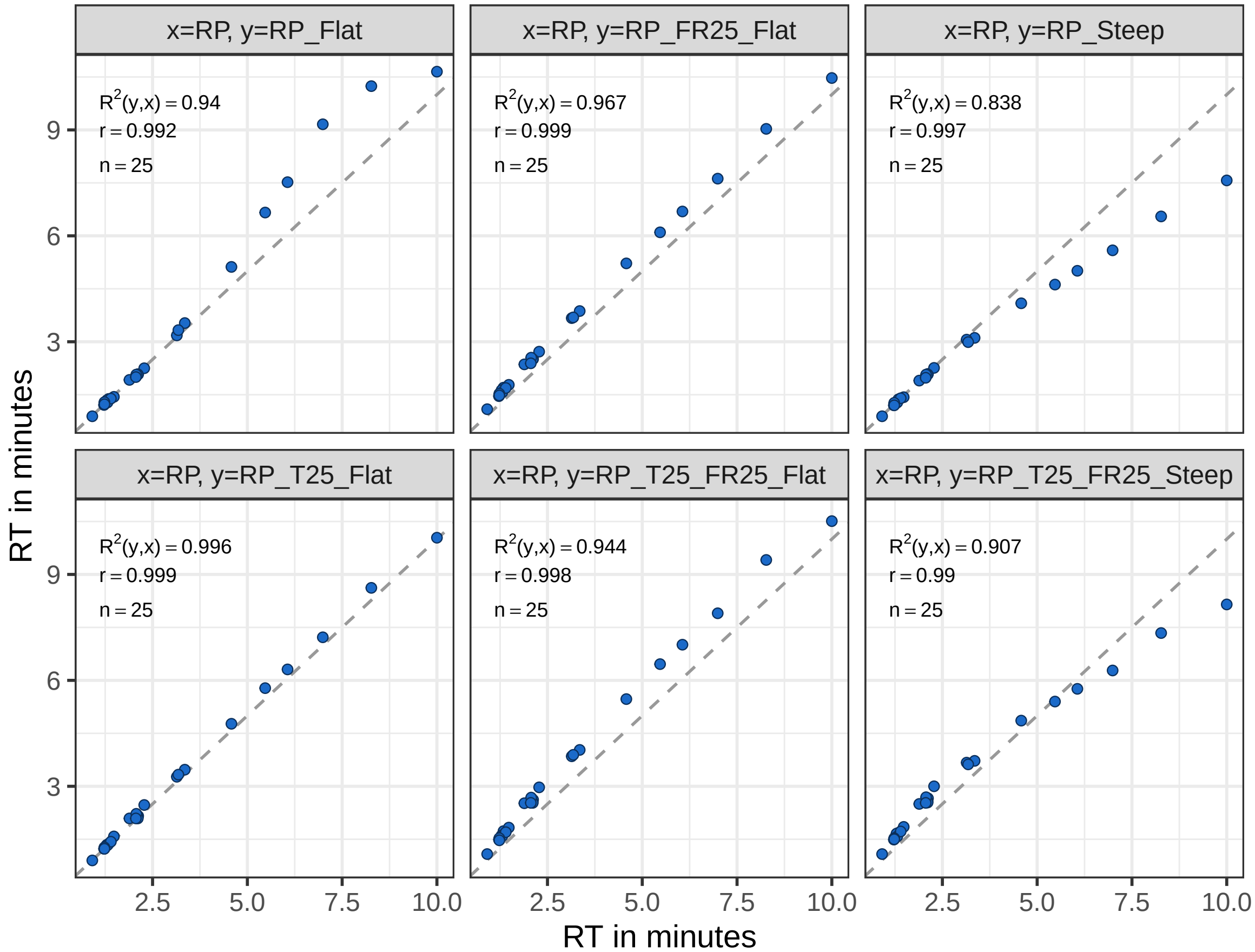

Figure S13: RT correlation for model adjustment across RP chromatographic variants. Each panel shows RTs from the base RP data set (x-axis) versus one of six variant RP data sets (y-axis), with all overlapping compounds plotted.

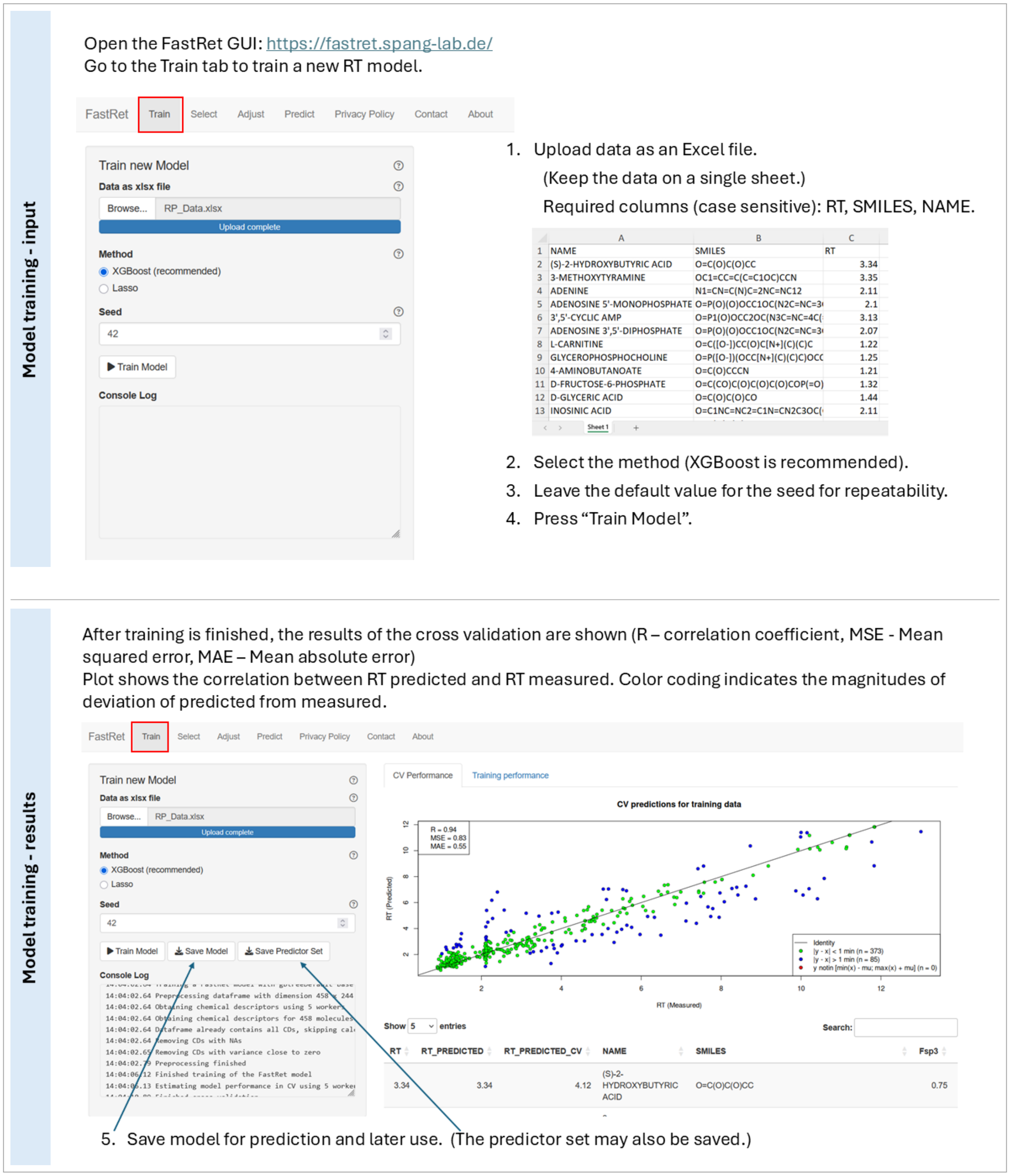

Figure S14: The *Train* tab of the FastRet web GUI. Upper slide: Users train a retention-time model from scratch by uploading an Excel file with the columns NAME, SMILES and RT (sidebar), choosing the model family (XGBoost or Lasso) and a random seed, and clicking *Train Model*. The seed defaults to a fixed value so that repeated training runs on the same data are reproducible. The always-visible console log below the controls reports progress in real time. Lower slide: After training, the main panel shows the cross-validation and training-set performance (measured vs. predicted RT, annotated with R^2, MSE and MAE) together with a table of the training compounds, their measured and predicted RTs and the computed molecular descriptors. The trained model and the predictor set can be downloaded for later use.

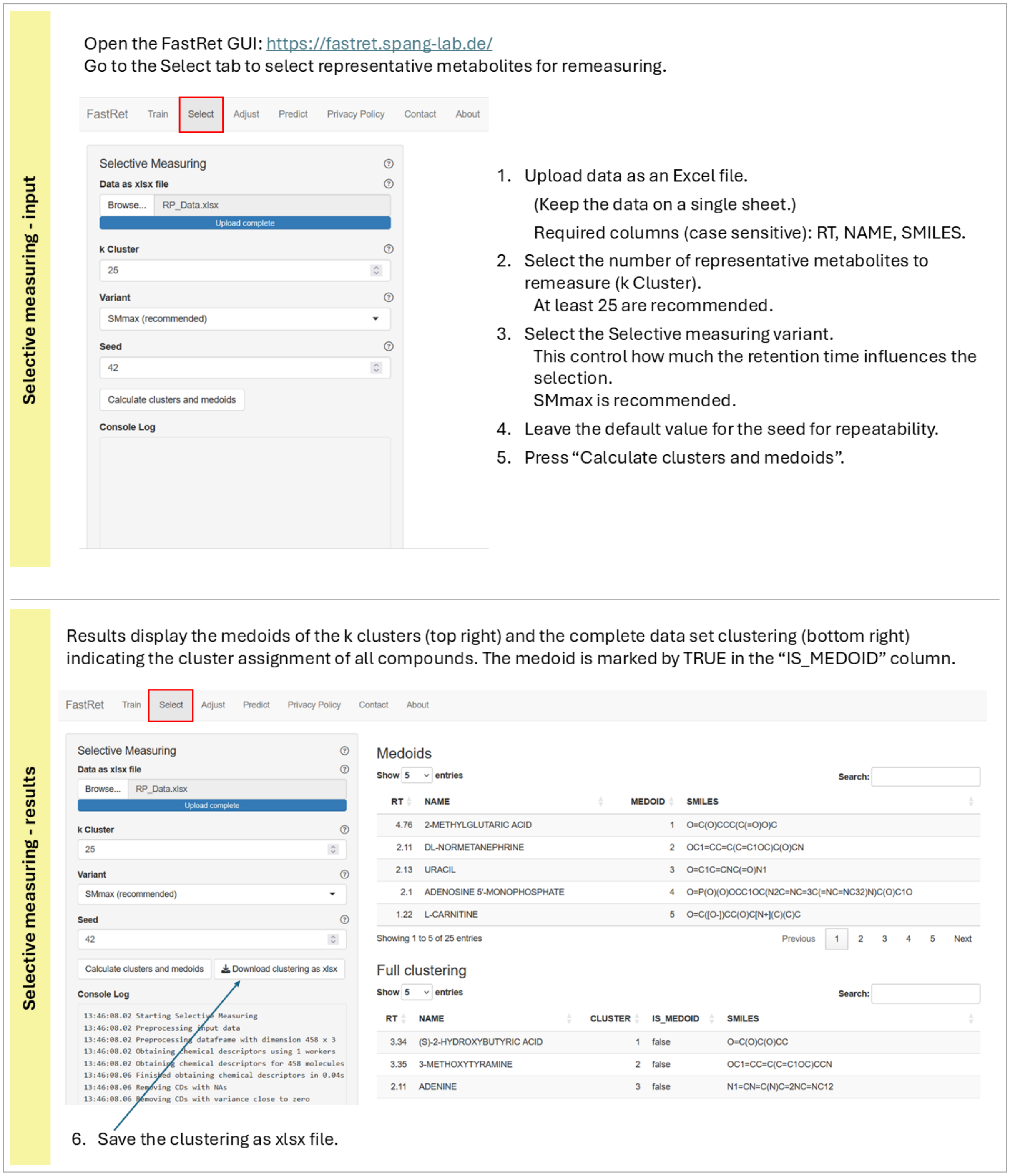

Figure S15: The *Select* (Selective Measuring) tab. Upper slide: Given an Excel file of candidate compounds (NAME, SMILES, RT), FastRet selects the k most representative molecules to be measured on a new chromatographic setup, using ridge-weighted PAM (k-medoids) clustering. Users set the desired number of clusters k and click *Calculate clusters and medoids*. Lower slide: The main panel then lists the selected medoids and the full cluster assignment, which can be exported as an Excel file. Measuring only these representatives keeps the experimental effort low while still covering the chemical diversity of the data set, providing an efficient basis for adjusting an existing model to the new setup.

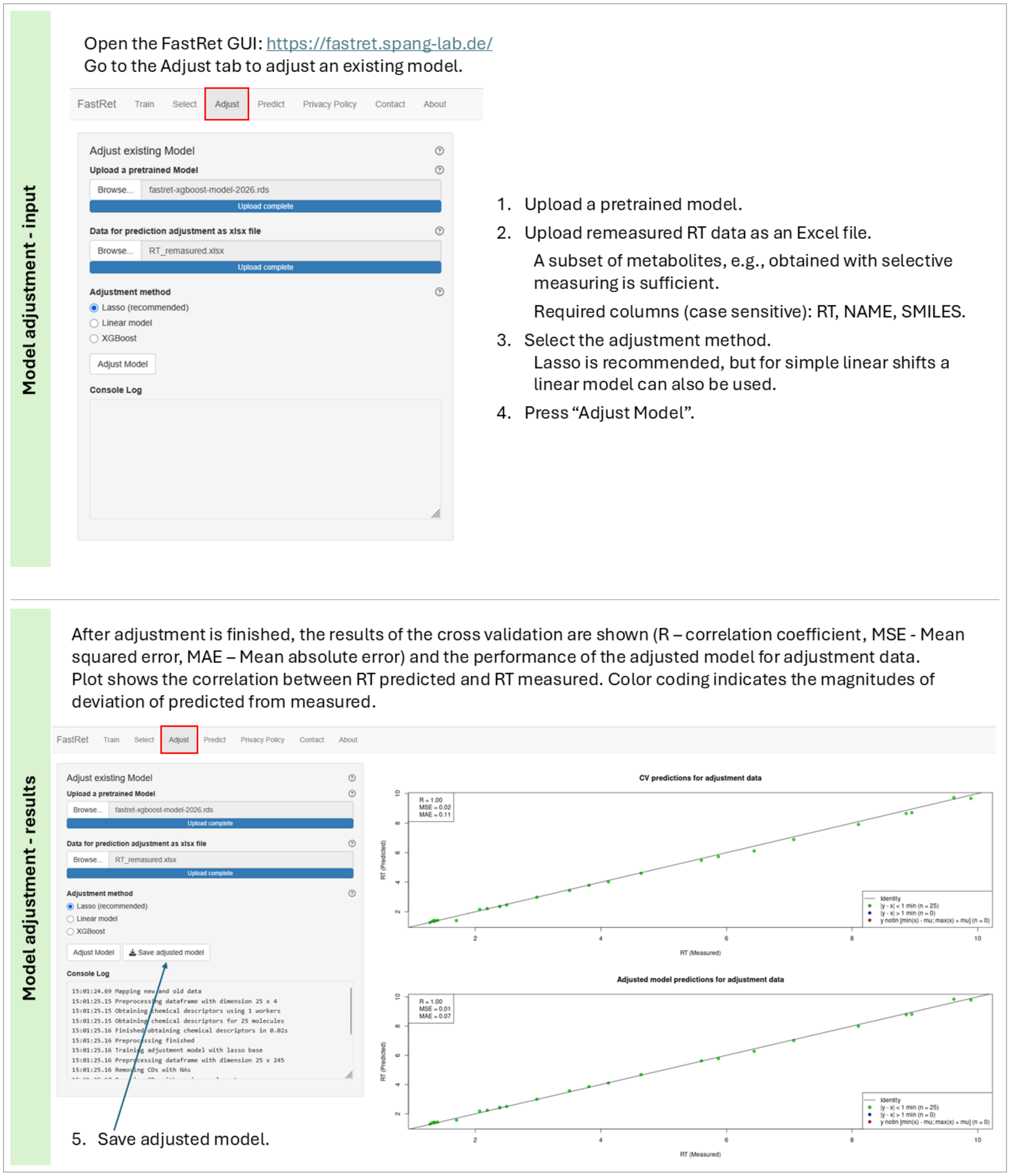

Figure S16: The *Adjust* tab. Upper slide: Users adapt an existing FastRet model to an altered chromatographic setup (e.g. changed temperature, pH or column age) by uploading the model and an Excel file of compounds remeasured under the new conditions (NAME, SMILES, RT). The *Adjustment method* selector chooses the family of the small correction model overlaid on the base model: Lasso (recommended) and XGBoost use the base RT together with the molecular descriptors and can therefore capture compound-specific shifts, whereas the linear model fits a simple straight-line correction from the base RT only. Lower slide: The main panel shows the cross-validation and adjusted-model performance, and the adjusted model can be downloaded and used like any other model.

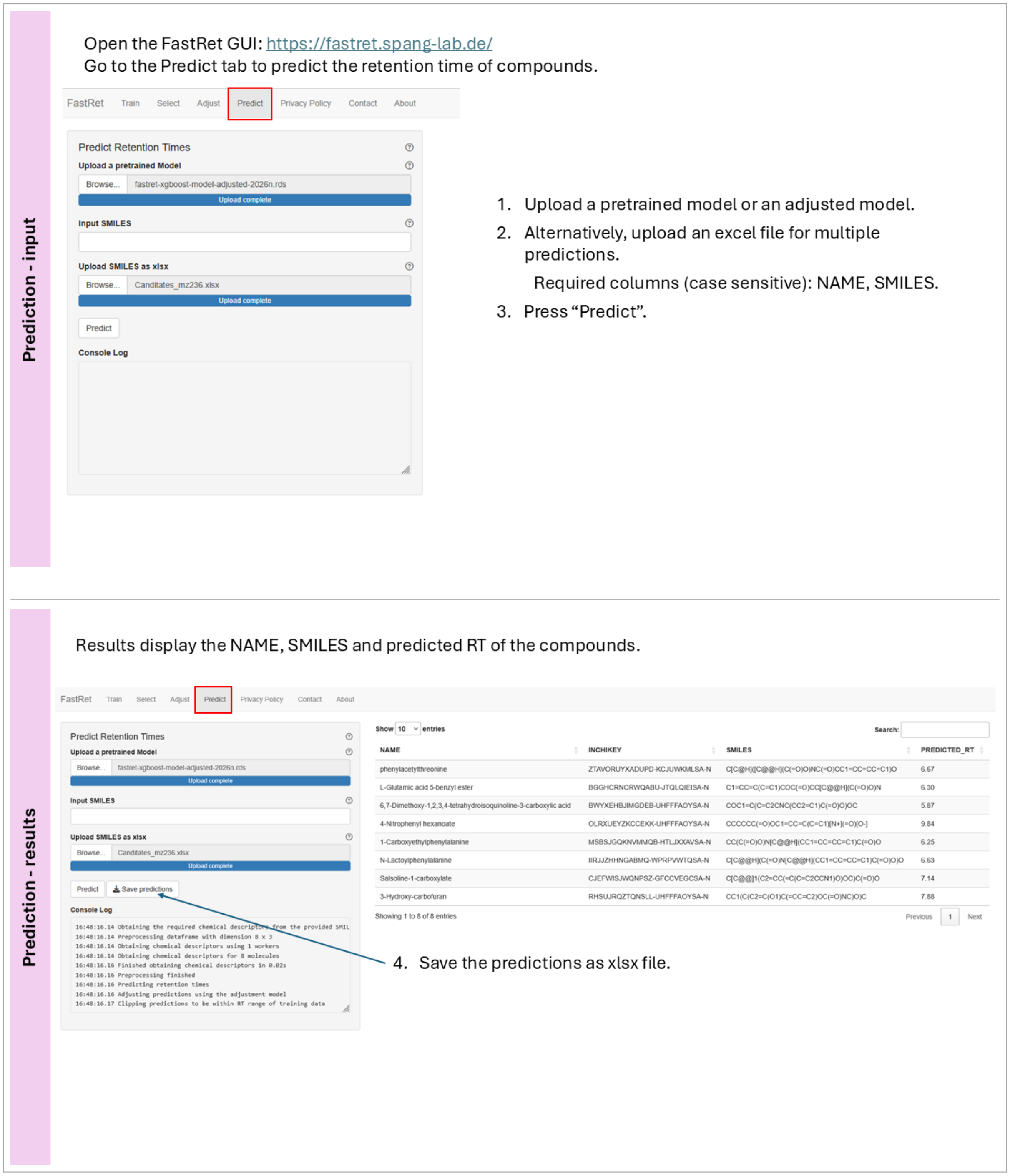

Figure S17: The *Predict* tab. Upper slide: Users apply a previously trained or adjusted model to new molecules. After uploading the model, retention times can be predicted either for a single compound entered as a SMILES string or for many compounds at once via an Excel file with the columns NAME and SMILES. Clicking *Predict* computes the retention times. Lower slide: Predicted retention times are shown in the main panel and can be exported as an Excel file.

50 µL of NIST SRM1905 plasma was extracted with methanol. Extract was evaporated to dryness and reconstituted in 100 µL pure water. Analysis by RP chromatography with ESI(-)QTOFMS.

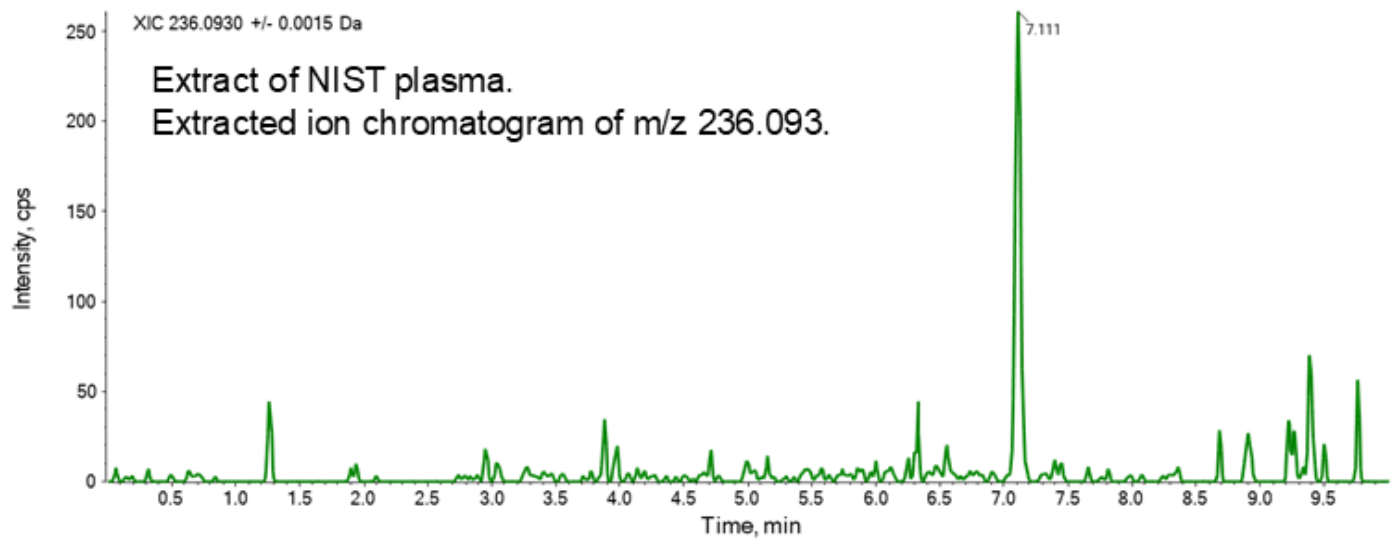

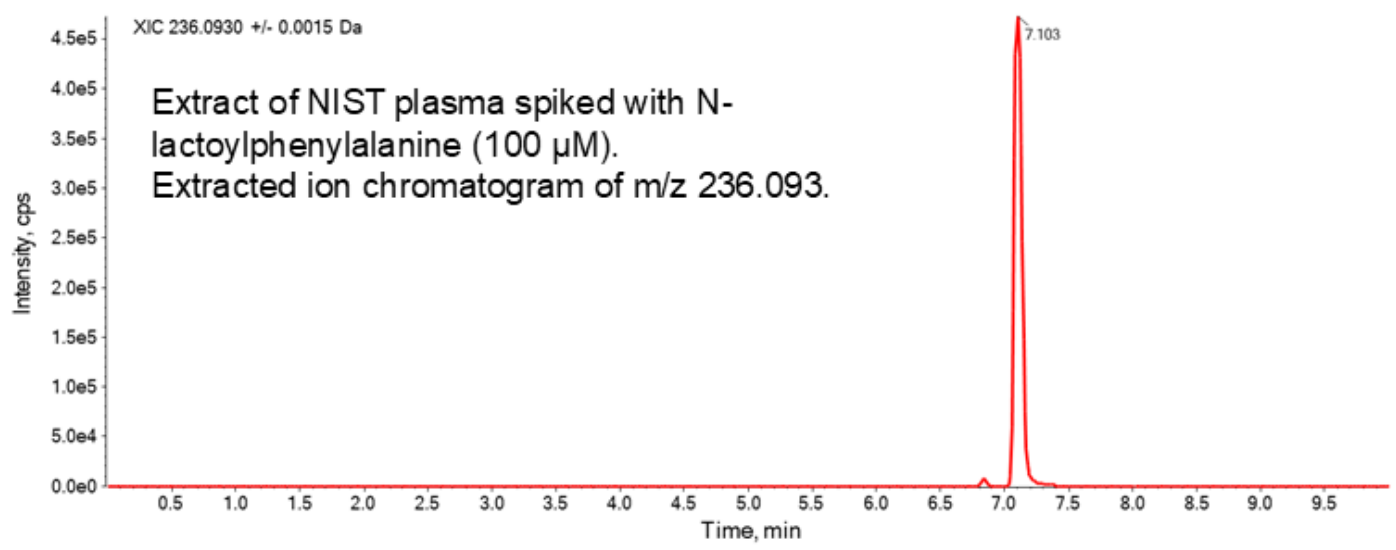

HMDB search results with predicted RT (RT_PRED).

NAME	FORMULA	[M-H]⁻	RT_PRED	|Δ RT |
**Phenylacetylthreonine**	**$C_{12}H_{15}NO_4$**	**236.0928**	**6.67**	**0.441**
L-Glutamic acid 5-benzyl ester	$C_{12}H_{15}NO_4$	236.0928	6.30	0.811
6,7-Dimethoxy-1,2,3,4-tetrahydroisoquinoline-3-carboxylic acid	$C_{12}H_{15}NO_4$	236.0928	5.87	1.241
4-Nitrophenyl hexanoate	$C_{12}H_{15}NO_4$	236.0928	9.84	2.729
1-Carboxyethylphenylalanine	$C_{12}H_{15}NO_4$	236.0928	6.25	0.861
**N-Lactoylphenylalanine**	**$C_{12}H_{15}NO_4$**	**236.0928**	**6.63**	**0.481**
**Salsoline-1-carboxylate**	**$C_{12}H_{15}NO_4$**	**236.0928**	**7.14**	**0.029**
3-Hydroxy-carbofuran	$C_{12}H_{15}NO_4$	236.0928	7.88	0.769

HMDB search of m/z 236.093, assuming an $[M-H]^-$ ion, yielded eight hits within a mass tolerance of 1 ppm. Three candidates exhibited a predicted RT within ± 0.5 min of the observed RT (7.11 min).

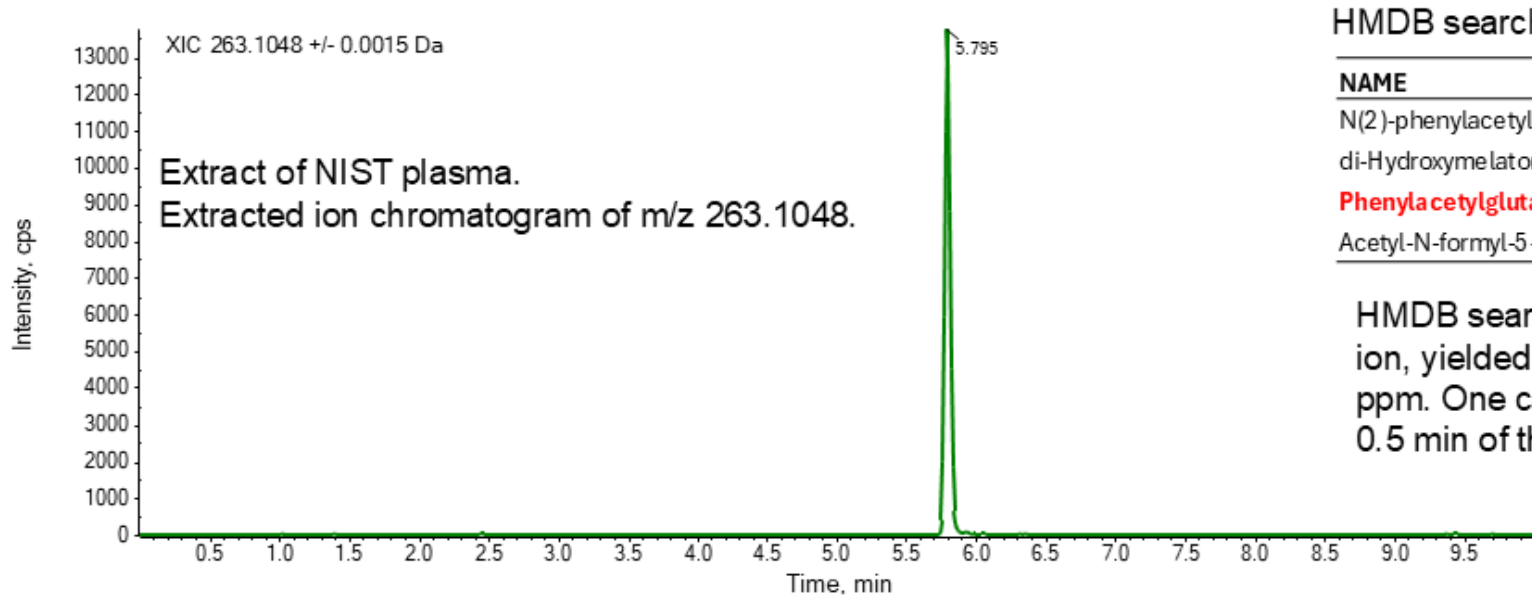

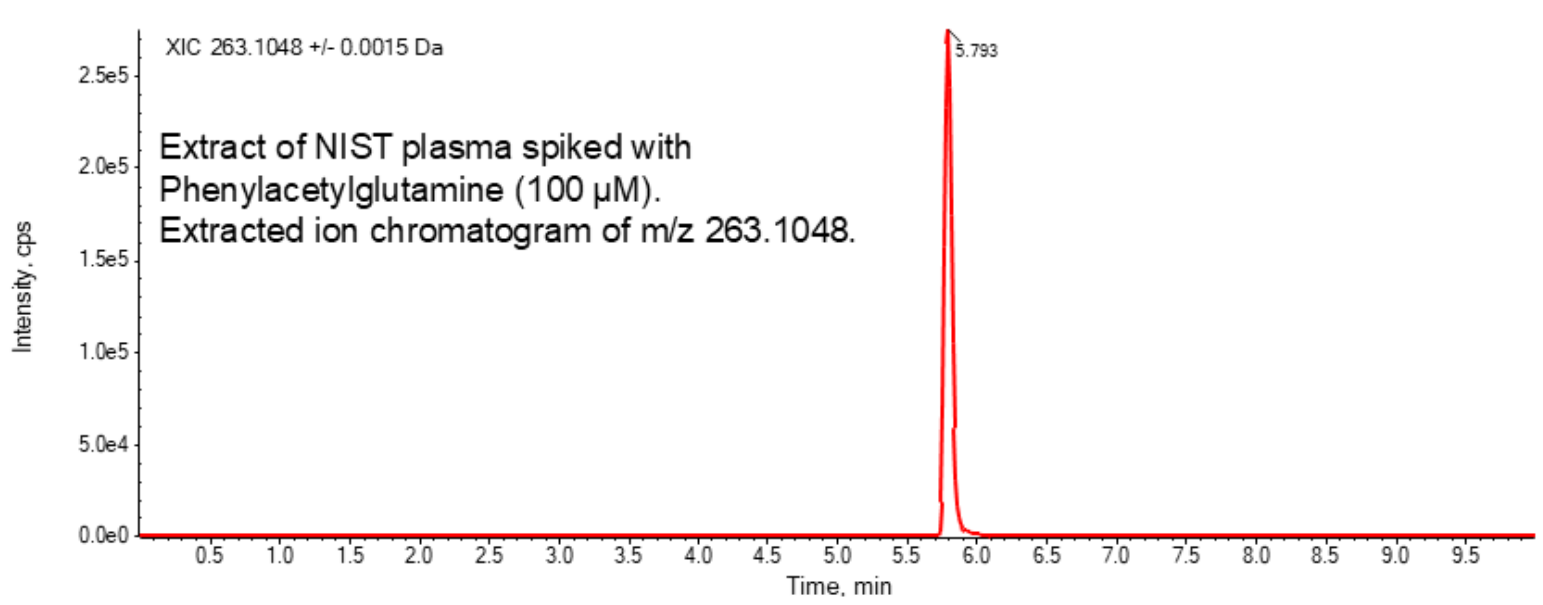

HMDB search results with predicted RT (RT_PRED).

NAME	FORMULA	[M-H]⁻	RT_PRED	|Δ RT |
N(2)-phenylacetyl-L-glutaminate	$C_{13}H_{16}N_2O_4$	263.1037	5.06	0.735
di-Hydroxymelatonin	$C_{13}H_{16}N_2O_4$	263.1037	7.46	1.665
**Phenylacetylglutamine**	**$C_{13}H_{16}N_2O_4$**	**263.1037**	**5.73**	**0.065**
Acetyl-N-formyl-5-methoxykynurenamine	$C_{13}H_{16}N_2O_4$	263.1037	6.65	0.855

HMDB search of m/z 263.1048, assuming an $[M-H]^-$ ion, yielded four hits within a mass tolerance of 4 ppm. One candidate exhibited a predicted RT within ± 0.5 min of the observed RT (5.795 min).

Figure S18: Application of FastRet. A NIST SRM1905 plasma extract was analyzed by RP-UHPLC-QTOFMS with electrospray ionization in negative mode. Two peaks at m/z 236.093 (upper slide) and m/z 263.1048 (lower slide) were examined. The accurate mass was searched in HMDB[1] and for the closest hits the RT was predicted. Upper slide: HMDB search of m/z 236.093, assuming an $[M-\mathrm{H}]^-$ ion, yielded eight hits within a mass tolerance of 1 ppm. Three candidates exhibited a predicted RT within ±0.5 min of the observed RT (7.11 min). Among those, N-lactoylphenylalanine was confirmed as the correct identification using an authentic standard spiked into the NIST plasma extract. Lower slide: HMDB search of m/z 263.1048, assuming an $[M-\mathrm{H}]^-$ ion, yielded four hits within a mass tolerance of 4 ppm. One candidate exhibited a predicted RT within ±0.5 min of the observed RT (5.795 min). This candidate, phenylacetylglutamine, was confirmed as the correct identification using an authentic standard spiked into the NIST plasma extract.

Tables

ID	Description
HSS-T3	ACQUITY Premier HSS T3 (Waters, 1.8 µm, 150 × 2.1 mm id) with VanGuard precolumn (Waters, 1.7 µm, 5 × 2.1 mm id)
BEH-AX	Atlantis PREMIER BEH C18 AX (Waters, 1.7 µm, 150 × 2.1 mm id) with VanGuard precolumn (Waters,1.7 µm, 5 × 2.1 mm id)
BEH-Amide	ACQUITY PREMIER BEH Amide (Waters, 1.7 µm, 150 × 2.1 mm id) with VanGuard precolumn (Waters, 1.7 µm, 5 × 2.1 mm id)
Retip[2]	ACQUITY PREMIER BEH Amide (Waters, 1.7 µm, 150 × 2.1 mm id) with VanGuard precolumn (1.7 µm, 5 × 2.1 mm id)

Table S1: Liquid chromatography columns used in this study. ID: column identifier. Description: phase type, manufacturer, particle size, length, and inner diameter of the column.

ID	Description
FA100-W	Formic Acid 0.1% (v/v) in Water
FA100-A	Formic Acid 0.1% (v/v) in ACN
FA100-AF10-W	Formic Acid 0.1% (v/v) and 10 mM Ammonium Formate in Water
FA125-AF10-W	Formic Acid 0.125% (v/v) and 10 mM Ammonium Formate in Water
FA100-AF10-WA10	Formic Acid 0.1% (v/v) and 10 mM Ammonium Formate in Water:ACN (10:90, v/v)
FA125-AF10-WA5	Formic Acid 0.125% (v/v) and 10 mM Ammonium Formate in Water:ACN (5:95, v/v)

Table S2: Mobile phase eluents (Eluent A and Eluent B) used in this study. ID: Eluent identifier. Description: Composition of the eluent, specifying solvent and additives with their concentrations.

ID	Description
Norm	3–50% in 8.0 min; 50–100% in 1.0 min; 100% for 3.5 min; 100–0% in 1.0 min; 0% for 8.5 min
NormB	0–50% in 8.0 min; 50–100% in 1.0 min; 100% for 3.5 min; 100–0% in 1.0 min; 0% for 8.5 min
Steep	3–50% in 5.0 min; 50–100% in 4.0 min; 100% for 3.5 min; 100–0% in 1.0 min; 0% for 8.5 min
Flat	3–30% in 8.0 min; 30–100% in 1.0 min; 100% for 3.5 min; 100–0% in 1.0 min; 0% for 8.5 min
AX	0–30% in 10.0 min; 30–100% in 5.0 min; 100% for 5.0 min; 100–0% in 2.0 min; 0% for 8.0 min
HILIC	97–70% in 8.0 min; 70–40% in 2.0 min; 40% for 1.5 min; 40–97% in 1.5 min; 97% for 6.0 min
Retip[2]	100% for 2 min ; 100–70% in 5.7 min; 70–40% in 1.8 min; 40–30% in 0.75 min; 30–100% in 2.5 min; 100% for 4.25 min

Table S3: Gradients used in this study in %B (percentage of eluent B in the mobile phase).

Nr	Name	RT[min]	Formula	PubChem CID
1	BIS(3-AMINOPROPYL)AMINE	1.09	C6H17N3	5942
2	L-CARNITINE	1.46	C7H15NO3	10917
3	L-GLUTAMINE	1.48	C5H10N2O3	5961
4	ALPHA-D-GLUCOSE	1.53	C6H12O6	79025
5	3-AMINOISOBUTANOATE	1.57	C4H9NO2	64956
6	ALPHA-D-GALACTOSE 1-PHOSPHATE	1.63	C6H13O9P	123912
7	N-ACETYLNEURAMINATE	1.69	C11H19NO9	439197
8	GLYCEROL 2-PHOSPHATE	1.7	C3H9O6P	2526
9	R-OCTOPAMINE	1.78	C8H11NO2	440266
10	(S)-MALATE	2.36	C4H6O5	222656
11	ADP-GLUCOSE	2.39	C16H25N5O15P2	16500
12	2'-DEOXYCYTIDINE 5'-MONOPHOSPHATE	2.5	C9H14N3O7P	13945
13	2'-DEOXYADENOSINE 5'-DIPHOSPHATE	2.51	C10H15N5O9P2	188966
14	UROCANATE	2.51	C6H6N2O2	736715
15	GLUTATHIONE	2.55	C10H17N3O6S	124886
16	HYPOXANTHINE	2.72	C5H4N4O	790
17	3-HYDROXY-3-METHYLGLUTARATE	3.67	C6H10O5	1662
18	INOSINE	3.69	C10H12N4O5	135398641
19	3-METHOXYTYRAMINE	3.87	C9H13NO2	1669
20	4-AMINOBENZOIC ACID	5.22	C7H7NO2	978
21	5-HYDROXYINDOLEACETATE	6.1	C10H9NO3	1826
22	3-HYDROXYPHENYLACETATE	6.69	C8H8O3	12122
23	FERULATE	7.62	C10H10O4	445858
24	SALICYLIC ACID	9.03	C7H6O3	338
25	CORTODOXONE	10.47	C21H30O4	440707

Table S4: Subset of 25 metabolites selected from an earlier version of Data set 1 using the SM1 selective measuring strategy and remeasured under different chromatographic conditions (summarized in Table 1) yielding Data sets 6–11. The exemplary RTs shown here were observed on the RP column using the “Norm” gradient (see Table S3).

Nr	Name	RT[min]	Formula	PubChem CID
1	3-METHYLHISTIDINE	1.08	C7H11N3O2	64969
2	GLYCYLSARCOSINE	1.3	C5H10N2O3	93131
3	ACEGLUTAMIDE	2.08	C7H12N2O4	182230
4	2-HYDROXYGLUTARIC ACID	2.18	C5H8O5	43
5	OXYPURINOL	2.86	C5H4N4O2	135398752
6	ALLOPURINOL	3.02	C5H4N4O	135401907
7	1-METHYLURIC ACID	3.43	C6H6N4O3	69726
8	LEVULINIC ACID	3.65	C5H8O3	11579
9	3-HEXENEDIOIC ACID	4.36	C6H8O4	5351896
10	ALPHA-HYDROXYHIPPURIC ACID	4.8	C9H9NO4	450272
11	PHLORETIC ACID	5.2	C9H10O3	10394
12	VANILLACTIC ACID	5.2	C10H12O5	160637
13	2,2-DIMETHYLSUCCINIC ACID	5.35	C6H10O4	11701
14	PHENYL HYDROGEN SULFATE	5.45	C6H6O4S	74426
15	N-ACETYL-3-MERCAPTO-DL-VALINE	5.55	C7H13NO3S	27372
16	1-METHYL-L-TRYPTOPHAN	5.6	C12H14N2O2	676159
17	PHENYLAC-GLN-OH	5.62	C13H16N2O4	92258
18	3-(INDOL-3-YL) LACTATE	7.1	C11H11NO3	676157
19	INDOLE-3-CARBOXALDEHYDE	7.69	C9H7NO	10256
20	3-INDOLEPROPIONIC ACID	8.9	C11H11NO2	3744
21	DICLOFENAC	10.8	C14H11Cl2NO2	3033
22	N-LAUROYLSARCOSINE	11.3	C15H29NO3	7348

Table S5: Validation set of 22 new metabolites measured under the same chromatographic conditions as Data sets 6–11 (summarized in Table 1), yielding Data sets 12–18. The exemplary RTs shown here were observed on the RP column using the “Norm” gradient (see Table S3).

**Descriptor (pattern)**	**Description**
MW, AMR	Molecular weight; molar refractivity.
XLogP, ALogP, ALogp2, MLogP	Predicted octanol–water partition coefficient (lipophilicity) from different models; ALogp2 is ALogP squared.
TopoPSA, tpsaEfficiency	Topological polar surface area, and TPSA divided by molecular weight.
nHBDon, nHBAcc	Number of hydrogen-bond donors / acceptors.
nRotB, nAtom, nB, nAtomP, nAtomLC, nAromBond, naAromAtom, nAcid, nBase	Simple counts: rotatable bonds, atoms, bonds, atoms in the largest π-system / longest chain, aromatic bonds / atoms, and acidic / basic groups.
nSmallRings, nAromRings, nRingBlocks, nAromBlocks, nRings3–nRings9	Ring counts by size, aromaticity and fusion.
Fsp3, HybRatio	Fraction of sp^3 carbons; ratio of sp^3 to sp^2 carbons.
C1SP1–C4SP3	Number of carbons of a given hybridization (SP1–SP3) bonded to 1–4 other carbons.
khs.$*$ (e.g. khs.sCH3, khs.aaCH, khs.ssO)	Kier–Hall electrotopological-state atom-type counts: the number of atoms matching a specific bonding environment (s = single, d = double, t = triple, a = aromatic bonds; element symbol gives the atom).
Kier1–Kier3	Kier κ molecular-shape indices (1st–3rd order).
SP.n/VP.n, SC.n/VC.n, SCH.n/VCH.n, SPC.n/VPC.n	Kier–Hall connectivity (chi) indices of order n for paths, clusters, chains and path-clusters; S = simple, V = valence-weighted.
WTPT.1–WTPT.5	Weighted-path (molecular-ID) descriptors.
WPATH, WPOL, Zagreb, VAdjMat, ECCEN	Classical topological indices: Wiener path and polarity numbers, Zagreb index, vertex-adjacency magnitude, eccentric connectivity.
MDEC.xy, MDEO.xy, MDEN.xy	Molecular distance-edge descriptors between carbon (C), oxygen (O) and nitrogen (N) atoms of primary–quaternary classes x,y.
ATSp/m/c1–5	Moreau–Broto autocorrelation of the molecular graph at topological lag 1–5, weighted by polarizability (p), mass (m) or charge (c).
BCUTw/c/p.1l, .1h	BCUT descriptors: lowest (l) and highest (h) Burden-matrix eigenvalues weighted by atomic weight (w), charge (c) or polarizability (p).
apol, bpol	Sum of atomic polarizabilities; sum of absolute bond-polarizability differences.
topoShape, geomShape, PetitjeanNumber	Petitjean topological and geometric molecular-shape indices.
fragC, FMF, LipinskiFailures	Fragment complexity; fraction of the molecule in its Murcko framework; number of Lipinski rule-of-five violations.
nA, nR, nN, . . . , nW	Amino-acid residue counts (peptide composition, one-letter codes); zero for non-peptide metabolites and therefore removed during filtering.

Table S6: Families of the 241 CDK descriptors used by FastRet, computed with `rcdk`. Patterns use wildcards (e.g. khs.$*$); n, x, y denote integer indices or atom classes. Descriptions summarize each family rather than listing every individual descriptor and follow the CDK descriptor documentation.[3,4]

Comparison	Data set	Mean ΔR^2	95% CI	p (Holm)
BRT − Lasso (from scratch)	RP	0.04	[0.02, 0.06]	<0.001
	RP-AXMM	0.29	[0.20, 0.37]	<0.001
	HILIC	0.10	[0.05, 0.15]	<0.001
SM1 − random (from scratch)	RP	0.10	[0.09, 0.11]	<0.001
	RP-AXMM	0.39	[0.37, 0.41]	<0.001
	HILIC	0.08	[0.07, 0.08]	<0.001
BRT–Lasso adj. − BRT (from scratch)	RP	0.31	[0.29, 0.33]	<0.001
	RP-AXMM	0.39	[0.37, 0.41]	<0.001
	HILIC	0.16	[0.15, 0.17]	<0.001

Table S7: Pairwise method comparisons over the 1000 shared seeds for models trained with 25 molecules. Each row reports the mean paired difference in test-set R^2 (same seed = same train/test split for both methods), its 95% confidence interval and the p-value of a Holm-adjusted paired t-test. Positive values favour the first method (BRT, SM1, or the adjusted model, respectively). Across all three data sets, BRT outperforms Lasso, selective measuring outperforms random drawing, and adjusted models outperform models trained from scratch. The mean is reported rather than the median because the R^2-difference distributions are skewed by occasional Lasso failures on unrepresentative draws, so the mean credits the more robust method.

Component	Main experiments	Benchmark workstation
Hardware	MacBook Pro, Apple M2 Max, 12 cores (8P + 4E), 32 GB RAM	2 × Intel Xeon Gold 6348, 56 cores / 112 threads, 1 TB RAM, no GPU
Operating system	macOS (Apple silicon)	Ubuntu 22.04
Platform	`aarch64-apple-darwin20`	`x86_64-pc-linux-gnu`
R	4.4.3	4.5.3
Java (for rcdk)	OpenJDK 11.0.29	OpenJDK 11.0.30
FastRet	1.3.5	1.4.4
glmnet (Lasso)	4.1-10	4.1-10
xgboost (BRT)	1.7.11.1	1.7.11.1
rcdk	3.8.1	3.8.1
rcdklibs	2.9	2.9
cluster (PAM)	2.1.8	2.1.8
future	1.68.0	1.67.0
Retip	—	2.0
scam (PredRet-like)	—	1.2.22
DeepGCN-RT	—	SMRT-pretrained; Python 3.10, PyTorch 2.2.2, DGL 1.1.0, dgllife 0.3.2 (CPU)

Table S8: Software and hardware environments for the reported experiments, as reported by `sessionInfo()`. Column *Main experiments* describes the machine used for descriptor computation, model training, the performance grid and Table S9; column *Benchmark workstation* describes the machine used for the external-method comparison of Table S11, on which all methods were timed on identical hardware. No specific macOS release is stated because those experiments spanned several operating-system updates. Package versions are given in CRAN form.

Method	CV Perf. Est.	Mean [s]	SD [s]
BRT	No	0.9611	0.3250
BRT	Yes	2.7523	0.3361
Lasso	No	0.8300	0.0498
Lasso	Yes	2.6365	0.1920

Table S9: Observed training runtimes for FastRet models on the RP data set. Runtimes were measured by training a FastRet model of type `method` with and without performance estimation in CV. Performance estimation used 5 folds and 5 worker processes. Each runtime benchmark was repeated 100 times with different random seeds; the table reports the resulting mean and standard deviation. Benchmarks were run with `verbose = 0` on the MacBook Pro laptop (Table S8).

**Model**	**Data set**	**Random**	**SMinf**	**SM0**	**SMmax**	**SM1**
Lasso	RP	0.48±0.29	-0.74±0.10	**0.61±0.10**	0.33±0.03	-0.19±0.02
	RP-AXMM	**-0.17±1.28**	-2.89±1.42	-0.55±0.28	-1.49±0.26	-2.34±0.26
	HILIC	0.41±0.80	0.42±0.10	0.55±0.09	0.56±0.20	**0.64±0.18**
BRT	RP	0.51±0.19	**0.75±0.01**	0.53±0.03	0.62±0.03	0.61±0.03
	RP-AXMM	0.11±0.31	0.47±0.03	0.25±0.24	0.32±0.20	**0.50±0.04**
	HILIC	0.50±0.14	0.46±0.03	0.55±0.07	0.55±0.04	**0.58±0.06**
Lasso-Identity	RP	0.05±0.04	0.05±0.04	0.05±0.04	0.05±0.04	0.05±0.04
	RP-AXMM	0.23±0.01	0.23±0.01	0.23±0.01	0.23±0.01	0.23±0.01
	HILIC	0.03±0.14	0.03±0.14	0.03±0.14	0.03±0.14	0.03±0.14
BRT-Identity	RP	0.30±0.00	0.30±0.00	0.30±0.00	0.30±0.00	0.30±0.00
	RP-AXMM	0.24±0.00	0.24±0.00	0.24±0.00	0.24±0.00	0.24±0.00
	HILIC	0.67±0.00	0.67±0.00	0.67±0.00	0.67±0.00	0.67±0.00
Lasso-Lm	RP	**0.76±0.01**	0.76±0.01	0.76±0.01	0.75±0.01	0.75±0.01
	RP-AXMM	0.41±0.08	0.39±0.01	0.46±0.01	**0.46±0.01**	0.36±0.01
	HILIC	0.20±0.16	**0.33±0.09**	0.11±0.13	0.08±0.14	0.05±0.15
BRT-Lm	RP	0.87±0.00	**0.87±0.00**	0.87±0.00	0.86±0.00	0.86±0.00
	RP-AXMM	0.50±0.05	0.53±0.00	0.53±0.00	**0.54±0.00**	0.54±0.00
	HILIC	0.69±0.01	**0.70±0.00**	0.70±0.00	0.70±0.00	0.70±0.00
Lasso-Lasso	RP	0.72±0.34	0.76±0.11	**0.77±0.01**	0.76±0.01	0.73±0.10
	RP-AXMM	0.43±0.14	0.19±0.06	0.48±0.04	**0.52±0.04**	0.31±0.02
	HILIC	**0.39±0.25**	0.16±0.10	0.27±0.11	0.28±0.10	0.25±0.10
BRT-Lasso	RP	0.82±0.33	0.86±0.08	**0.87±0.00**	0.87±0.00	0.86±0.04
	RP-AXMM	0.50±0.15	0.22±0.06	0.49±0.04	**0.52±0.04**	0.42±0.02
	HILIC	0.66±0.19	0.63±0.05	0.69±0.09	**0.70±0.03**	0.69±0.02
Lasso-BRT	RP	0.76±0.04	0.77±0.00	0.77±0.01	0.78±0.01	**0.79±0.01**
	RP-AXMM	0.24±0.25	0.48±0.02	0.43±0.22	0.37±0.17	**0.57±0.09**
	HILIC	0.69±0.08	0.70±0.03	**0.75±0.01**	0.75±0.01	0.73±0.01
BRT-BRT	RP	0.82±0.06	0.85±0.00	**0.87±0.00**	0.85±0.00	0.86±0.00
	RP-AXMM	0.26±0.24	0.50±0.03	0.39±0.20	0.42±0.16	**0.56±0.04**
	HILIC	0.66±0.07	0.68±0.02	**0.70±0.02**	0.69±0.01	0.67±0.01

Table S10: R^2 (mean ± SD) over 1000 seeds at $n_{\text{train}} = 25$. For hyphenated model names, the part before the hyphen is the base model and the part after the hyphen is the adjustment model; unhyphenated names denote models trained from scratch. The best value per row is written in bold. SM1 is most effective for training from scratch and for BRT adjustment; SMmax is best for Lasso adjustment; SMinf is best for Lm adjustment.

Tool	Setting	n	Cores	t_{train} (s)	t_{predict} (s)
FastRet-BRT	TFS	full	1	1.60 ± 0.47	0.39 ± 0.05
FastRet-Lasso	TFS	full	1	2.16 ± 0.51	0.43 ± 0.02
FastRet-BRT-GS	TFS	full	8	13.93 ± 3.56	0.41 ± 0.05
Retip-xgboost	TFS	full	16	47.34 ± 32.18	0.01 ± 0.00
Retip-keras	TFS	full	16	86.74 ± 49.69	0.31 ± 0.05
DeepGCN-RT	TFS	full	16	5074.74 ± 1492.74	1.20 ± 0.56
FastRet-BRT-LM	Adj	25	1	0.00 ± 0.00	0.42 ± 0.03
PredRet-like	Adj	25	1	0.03 ± 0.01	0.00 ± 0.00
FastRet-BRT-Lasso	Adj	25	1	0.49 ± 0.03	0.42 ± 0.03
FastRet-TFS25-Lasso	Adj	25	1	0.50 ± 0.03	0.42 ± 0.02
FastRet-TFS25-BRT	Adj	25	1	0.54 ± 0.03	0.42 ± 0.03
Retip-xgboost	Adj	25	16	12.85 ± 2.63	0.01 ± 0.00
Retip-keras	Adj	25	16	29.38 ± 6.18	0.28 ± 0.02
DeepGCN-RT	Adj	25	16	429.46 ± 106.20	1.36 ± 0.81
FastRet-BRT-LM	Adj	50	1	0.00 ± 0.00	0.41 ± 0.04
PredRet-like	Adj	50	1	0.03 ± 0.02	0.00 ± 0.00
FastRet-BRT-Lasso	Adj	50	1	0.50 ± 0.03	0.42 ± 0.03
FastRet-TFS25-Lasso	Adj	50	1	0.51 ± 0.04	0.40 ± 0.05
FastRet-TFS25-BRT	Adj	50	1	0.65 ± 0.06	0.43 ± 0.06
Retip-xgboost	Adj	50	16	23.05 ± 1.22	0.01 ± 0.00
Retip-keras	Adj	50	16	73.98 ± 26.83	0.40 ± 0.11
DeepGCN-RT	Adj	50	16	935.65 ± 278.84	1.52 ± 0.65

Table S11: Wall-clock training and prediction time on a dual Intel Xeon Gold 6348 workstation (Table S8), as mean ± SD over the three core data sets RP, RP-AXMM and HILIC and the benchmark seeds. Cores is the core count each tool was pinned to; FastRet and PredRet-like run single-core, the neural and graph methods use 16, and n is the training-set size. FastRet times use a warm descriptor cache, the realistic repeated-use case; Retip instead recomputes CDK descriptors on every call. DeepGCN-RT is slowest by two to three orders of magnitude.

Type	ID	Column	Source	n_{train}	n_{test}	From scratch	Adjust-25	Adjust-50
							test-set R^2	
RP	0048	Hypersil GOLD	PredRet	358	193	0.73	0.73	0.72
RP	0433	Ascentis Express C18	Bruderer	97	108	0.55	0.83	0.85
RP	0403	ACQUITY BEH C18	Han	312	204	0.77	0.33	0.64
RP	0392	ACQUITY Premier BEH C18	La Frano	309	354	0.79	0.84	0.85
RP	0400	ACQUITY HSS C18	Han	259	189	0.62	0.61	0.61
RP	0246	Kinetex XB-C18	BGC	191	372	0.06	0.54	0.43
RP	0238	ACQUITY HSS T3	BGC	180	371	0.42	-2.08	0.54
RP	0435	XSelect HSS T3 XP	Bruderer	168	188	-0.36	-0.06	-0.07
RP	0318	CORTECS C18	BGC	166	327	0.58	0.68	0.69
RP	0329	Raptor Biphenyl	BGC	163	229	0.68	0.57	0.65
RP	0062	Kinetex EVO C18	PredRet	104	132	0.10	0.25	0.22
RP	0438	ACQUITY Premier HSS T3	n/a	90	108	0.68	0.83	0.79
RP		**Median**				**0.60**	**0.59**	**0.65**
HILIC	0228	ACQUITY BEH Amide	PredRet	176	812	0.60	0.58	0.57
HILIC	0183	ACQUITY BEH Amide	Pezzatti	219	62	0.56	0.49	0.53
HILIC		**Median**				**0.58**	**0.53**	**0.55**

Table S12: Cross-lab external validation of FastRet on RepoRT data sets.[5] RepoRT is a public repository that provides more than 400 retention time data sets from many labs.[5] We used 12 RP columns from five manufacturers and 2 HILIC columns, measured on different instruments in different labs. We kept a RepoRT column only if it shared at least 50 molecules with our in-house RP or HILIC library and still left at least 100 test molecules for RP or 50 test molecules for HILIC. Each data set was split into a training set and a test set. Molecules that also occur in our in-house library were put into the training set. The remaining molecules formed the test set. Compounds were matched by InChIKey. This way our SMmax approach could pick any training molecule for adjustment, and no test molecule overlaps with our library. n_{train} and n_{test} are the numbers of training and test molecules. For each data set we trained and evaluated FastRet only on that data set's own molecules and retention times. We report the test-set R^2 of three settings. From scratch is a FastRet-BRT model trained on the training molecules. Adjust-25 and Adjust-50 take our in-house RP or HILIC BRT model and adapt it to the new column with a Lasso adjustment. They use 25 or 50 training molecules chosen by SMmax. All three settings are scored on the same test molecules. The Median rows give the median R^2 per separation mode. ID is the RepoRT data set identifier. Source is the data origin listed in RepoRT. It names the first author of the original publication or the lab that contributed the data set. All numbers come from a single run with seed 42.

Listings

```r
getCDs ← function(smiles) {
    cds ← load_cached_descriptors()
    new_smiles ← unique(smiles[smiles %notin% rownames(cds)])
    if (length(new_smiles) > 0) {
        mols ← rcdk::parse.smiles(new_smiles)
        new_cds ← rcdk::eval.desc(mols, CDNames)
        cds ← update_cache(cds, new_smiles, new_cds)
    }
    cds[smiles, ]
}
```

Listing S1: Procedure for computing chemical descriptors for a list of SMILES strings. Descriptors for known compounds are loaded from cache; only new SMILES are evaluated with `rcdk`. This is a simplified version of the full implementation; the full code is available at `https://github.com/spang-lab/fastret`.

```r
train_frm <- function(df, method) {
    X <- getCDs(df$SMILES)
    y <- df$RT
    if (method == "Lasso") {
        model <- train_glmnet(
            X, y, alpha = 1, standardize = TRUE, family = "gaussian",
            type.measure = "mse", lambda = "lambda.min"
        )
    } else if (method == "BRT") {
        model <- train_xgboost(
            X, y, params = list(objective = "reg:squarederror", nthread = 1),
            nrounds = 2000, nfold = 10, early_stopping_rounds = 20
        )
    }
}
```

Listing S2: Procedure for training a machine learning model from an input data set `df`. Mandatory columns of `df` are `SMILES`, `RT` and `NAME`. Argument `method` specifies the machine learning algorithm to use, which can be either `Lasso` or `BRT`. This is a simplified version of the full implementation; the full code is available at `https://github.com/spang-lab/fastret`.

```r
selective_measuring <- function(X, k, rt_coef) {
    Z     <- standardize(X)
    model <- fit_ridge(Z, y = "RT")
    S     <- scale_by_coefs(Z, model, rt_coef)
    meds  <- k_medoids(S, k)
    meds
}
```

Listing S3: Procedure for selecting `k` metabolites to remeasure from a base data set `X`. Parameter `rt_coef` controls the influence of RT during clustering. This is a simplified version of the full implementation; the full code is available at `https://github.com/spang-lab/fastret`.

```r
adjust_frm <- function(base_model, new_data, adj_type) {
    X <- getCDs(new_data$SMILES)
    base_data <- get_training_data(base_model)
    X$RT <- base_data$RT[match(new_data$INCHIKEY, base_data$INCHIKEY)]
    y <- new_data$RT
    base_model$adj$model <- train_model(y, X, adj_type)
}
```

Listing S4: Procedure for adjusting the base model using `new_data` and an `adj_type` model. The fitting procedure is the same as for training from scratch, except that the adjustment model uses chemical descriptors from `new_data` together with RTs from the base data set, matched by `INCHIKEY`. This is a simplified version of the full implementation; the full code is available at `https://github.com/spang-lab/fastret`.

```r
clip_predictions <- function(yhat, y) {
    log_y <- log(y)
    mu <- mean(log_y)
    sigma <- stats::sd(log_y)
    lower_bound <- stats::qlnorm(5e-04, meanlog = mu, sdlog = sigma)
    upper_bound <- stats::qlnorm(0.9995, meanlog = mu, sdlog = sigma)
    obsrvd_min <- min(y)
    obsrvd_max <- max(y)
    if (lower_bound > obsrvd_min) lower_bound <- obsrvd_min
    if (lower_bound < obsrvd_min * 0.01) lower_bound <- obsrvd_min * 0.01
    if (upper_bound < obsrvd_max) upper_bound <- obsrvd_max
    yhat <- pmin(yhat, upper_bound)
    yhat <- pmax(yhat, lower_bound)
    yhat
}
```

Listing S5: Procedure for clipping predicted RTs $\hat{y}$ using the observed RTs y. This is a simplified version of the full implementation; the full code is available at `https://github.com/spang-lab/fastret`.

```r
predict.frm <- function(frm, df, clip=TRUE) {
    X <- getCDs(df)
    X2 <- impute_missing_predictors(X, frm$model, frm$df)
    yhat <- predict(frm$model, X2, clip)
    if (is.null(frm$adj$model)) return(yhat)
    X2 <- impute_missing_predictors(X, frm$adj$model, frm$adj$df)
    X2$RT <- yhat
    yhat <- predict(frm$adj$model, X2, clip)
}
```

Listing S6: Procedure for predicting RTs from a FastRet model for a new data set `df`. If 'clip' is TRUE, RT predictions are clipped to be in a sensible range, determined from the training data. For adjusted models, the base model's RT predictions are used as input to the adjustment model. This is a simplified version of the full implementation; the full code is available at `https://github.com/spang-lab/fastret`.

```r
estimate_performance <- function(
    dcnr, base_type, mod_type, sel_meth, n_train, seed
) {
    set.seed(seed)
    XX <- get_datasets(dcnr)
    X_train <- select_measurements(XX$train, n_train, sel_meth)
    if (base_type == "None") {
        final_model <- train_frm(X_train, mod_type)
    } else {
        base_model  <- train_frm(XX$base, base_type)
        final_model <- adjust_frm(base_model, X_train, mod_type)
    }
    RT_pred <- predict(final_model, XX$test)
    stats <- calculate_error_metrics(XX$test$RT, RT_pred)
}
```

Listing S7: Procedure for estimating the performance of a FastRet model of type `mod_type`, trained on `n_train` metabolites selected using method `sel_meth`. Which data set is used as Base, Train and Test is determined by the data set combination number `dcnr` (see Table 2). The type of the base model is determined by `base_type`, which can be `Lasso`, `BRT`, or `None` (indicating training from scratch). Parameter `seed` is used to ensure reproducibility of all stochastic parts of the procedure. This is a simplified version of the full implementation; the full code is available at `https://github.com/spang-lab/fastret`.

```
split_datasets <- function() {
    shared <- intersect(HILIC$INCHIKEY, HILIC_Retip$INCHIKEY)
    HILIC_Train       <- HILIC[HILIC$INCHIKEY %in% shared, ]
    HILIC_Test        <- HILIC[HILIC$INCHIKEY %notin% shared, ]
    HILIC_Retip_Train <- HILIC_Retip[HILIC_Retip$INCHIKEY %in% shared, ]
    HILIC_Retip_Test  <- HILIC_Retip[HILIC_Retip$INCHIKEY %notin% shared, ]

    shared <- intersect(RP$INCHIKEY, RPB$INCHIKEY, RP_AXMM$INCHIKEY)
    train <- sample(shared, 300)
    RP_Train      <- RP[RP$INCHIKEY %in% train, ]
    RP_Test       <- RP[RP$INCHIKEY %notin% train, ]
    RPB_Train     <- RPB[RPB$INCHIKEY %in% train, ]
    RPB_Test      <- RPB[RPB$INCHIKEY %notin% train, ]
    RP_AXMM_Train <- RP_AXMM[RP_AXMM$INCHIKEY %in% train, ]
    RP_AXMM_Test  <- RP_AXMM[RP_AXMM$INCHIKEY %notin% train, ]

    train1 <- sample(train, 150)
    RP_Train1 <- RP[RP$INCHIKEY %in% train1, ]
    RPB_Train1 <- RPB[RPB$INCHIKEY %in% train1, ]
    RP_AXMM_Train1 <- RP_AXMM[RP_AXMM$INCHIKEY %in% train1, ]
}
```

Listing S8: Procedure for constructing data set combinations with paired base and training data sets with 100% overlap and distinct test data sets. This is a simplified version of the full implementation; the full code is available at `https://github.com/spang-lab/fastret`.

Supplementary Notes

Supplementary Note S1: Lasso

Lasso[6] extends multiple linear regression by introducing an L_1-penalty on the regression coefficients. This penalty encourages sparsity, shrinking less informative coefficients to zero and improving model generalization. The model coefficients β are determined by minimizing the following objective function with respect to β:

$$\sum_{i=1}^{n} \left(y_i - \beta_0 - \sum_{j=1}^{p} x_{ij}\, \beta_j \right)^2 \; + \; \lambda \sum_{j=1}^{p} |\beta_j|$$

where n denotes the number of training samples, p the number of features, x_{ij} the value of feature j for sample i, y_i the observed RT for sample i, and λ the regularization parameter.

Lasso model fitting was performed using the R package `glmnet`.[7–10] For each model, the optimal λ was selected from 100 candidate values using tenfold internal cross-validation.

Supplementary Note S2: Boosted Regression Trees

One particular problem we observed with Lasso is that it can produce extreme outliers when predicting metabolites with descriptor values outside the range present in the training data. Boosted Regression Trees (BRTs) avoid this issue because their predictions are naturally constrained to the range of RTs seen during training.

BRTs combine multiple weak learners, typically shallow decision trees, into an ensemble model. Each tree is trained to correct the prediction error of the current ensemble, using a squared error loss (RMSE).[11]

The R package `xgboost`[12] was used to train and tune the models. Hyperparameters were left at their default values, except for the `nrounds` parameter, which was optimized using an internal tenfold cross-validation.

References

(1) Wishart, D. S.; Guo, A.; Oler, E.; Wang, F.; Anjum, A.; Peters, H.; Dizon, R.; Sayeeda, Z.; Tian, S.; Lee, B. L.; Berjanskii, M.; Mah, R.; Yamamoto, M.; Jovel, J.; Torres-Calzada, C.; Hiebert-Giesbrecht, M.; Lui, V. W.; Varshavi, D.; Varshavi, D.; Allen, D.; Arndt, D.; Khetarpal, N.; Sivakumaran, A.; Harford, K.; Sanford, S.; Yee, K.; Cao, X.; Budinski, Z.; Liigand, J.; Zhang, L.; Zheng, J.; Mandal, R.; Karu, N.; Dambrova, M.; Schiöth, H. B.; Greiner, R.; Gautam, V. HMDB 5.0: The Human Metabolome Database for 2022. *Nucleic Acids Research* **2022**, *50*, D622–D631, DOI: `10.1093/nar/gkab1062`.

(2) Bonini, P.; Kind, T.; Tsugawa, H.; Barupal, D. K.; Fiehn, O. Retip: Retention Time Prediction for Compound Annotation in Untargeted Metabolomics. *Analytical Chemistry* **2020**, *92*, 7515–7522, DOI: `10.1021/acs.analchem.9b05765`.

(3) Willighagen, E. L.; Mayfield, J. W.; Alvarsson, J.; Berg, A.; Carlsson, L.; Jeliazkova, N.; Kuhn, S.; Pluskal, T.; Rojas-Chertó, M.; Spjuth, O.; Torrance, G.; Evelo, C. T.; Guha, R.; Steinbeck, C. The Chemistry Development Kit (CDK) v2.0: Atom Typing, Depiction, Molecular Formulas, and Substructure Searching. *Journal of Cheminformatics* **2017**, *9*, 33, DOI: `10.1186/s13321-017-0220-4`.

(4) Guha, R. rcdk: Interface to the CDK Libraries, R package version 3.8.1, 2023.

(5) Kretschmer, F.; Harrieder, E.-M.; Hoffmann, M. A.; Böcker, S.; Witting, M. RepoRT: a comprehensive repository for small molecule retention times. *Nature Methods* **2024**, *21*, 153–155, DOI: `10.1038/s41592-023-02143-z`.

(6) Hastie, T.; Tibshirani, R.; Friedman, J. H., *The Elements of Statistical Learning: Data Mining, Inference, and Prediction*, 2nd ed; Springer Series in Statistics; Springer: New York, NY, 2009.

(7) Friedman, J.; Tibshirani, R.; Hastie, T. Regularization Paths for Generalized Linear Models via Coordinate Descent. *Journal of Statistical Software* **2010**, *33*, 1–22, DOI: `10.18637/jss.v033.i01`.

(8) Simon, N.; Friedman, J.; Tibshirani, R.; Hastie, T. Regularization Paths for Cox's Proportional Hazards Model via Coordinate Descent. *Journal of Statistical Software* **2011**, *39*, 1–13, DOI: 10.18637/jss.v039.i05.

(9) Tay, J. K.; Narasimhan, B.; Hastie, T. Elastic Net Regularization Paths for All Generalized Linear Models. *Journal of Statistical Software* **2023**, *106*, 1–31, DOI: 10.18637/jss.v106.i01.

(10) Friedman, J.; Hastie, T.; Tibshirani, R.; Narasimhan, B.; Tay, K.; Simon, N.; Yang, J. glmnet: Lasso and Elastic-Net Regularized Generalized Linear Models, R package version 4.1-8, 2023.

(11) Schapire, R. E., The Boosting Approach to Machine Learning: An Overview. In *Nonlinear Estimation and Classification*, Bickel, P., Diggle, P., Fienberg, S., Krickeberg, K., Olkin, I., Wermuth, N., Zeger, S., Denison, D. D., Hansen, M. H., Holmes, C. C., Mallick, B., Yu, B., Eds.; Springer New York: New York, NY, 2003; Vol. 171, pp 149–171, DOI: 10.1007/978-0-387-21579-2_9.

(12) Chen, T.; Guestrin, C. XGBoost: A Scalable Tree Boosting System. *Proceedings of the 22nd ACM SIGKDD International Conference on Knowledge Discovery and Data Mining* **2016**, 785–794, DOI: 10.1145/2939672.2939785.
